# Methodological limitations in studies assessing the effects of environmental and socioeconomic variables on the spread of COVID-19: a systematic review

**DOI:** 10.1186/s12302-021-00550-7

**Published:** 2021-09-10

**Authors:** Maria A. Barceló, Marc Saez

**Affiliations:** 1 Research Group On Statistics, Econometrics and Health (GRECS), and CIBER of Epidemiology and Public Health (CIBERESP), University of Girona, Carrer de la Universitat de Girona 10, Campus de Montilivi, 17003 Girona, Spain; 2 CIBER of Epidemiology and Public Health (CIBERESP), Madrid, Spain

**Keywords:** COVID-19, Environmental (meteorological and air pollutants) variables, Socioeconomic variables, Social contacts

## Abstract

**Background:**

While numerous studies have assessed the effects of environmental (meteorological variables and air pollutants) and socioeconomic variables on the spread of the COVID-19 pandemic, many of them, however, have significant methodological limitations and errors that could call their results into question. Our main objective in this paper is to assess the methodological limitations in studies that evaluated the effects of environmental and socioeconomic variables on the spread of COVID-19.

**Main body:**

We carried out a systematic review by conducting searches in the online databases PubMed, Web of Science and Scopus up to December 31, 2020. We first excluded those studies that did not deal with SAR-CoV-2 or COVID-19, preprints, comments, opinion or purely narrative papers, reviews and systematic literature reviews. Among the eligible full-text articles, we then excluded articles that were purely descriptive and those that did not include any type of regression model. We evaluated the risk of bias in six domains: confounding bias, control for population, control of spatial and/or temporal dependence, control of non-linearities, measurement errors and statistical model. Of the 5631 abstracts initially identified, we were left with 132 studies on which to carry out the qualitative synthesis. Of the 132 eligible studies, we evaluated 63.64% of the studies as high risk of bias, 19.70% as moderate risk of bias and 16.67% as low risk of bias.

**Conclusions:**

All the studies we have reviewed, to a greater or lesser extent, have methodological limitations. These limitations prevent conclusions being drawn concerning the effects environmental (meteorological and air pollutants) and socioeconomic variables have had on COVID-19 outcomes. However, we dare to argue that the effects of these variables, if they exist, would be indirect, based on their relationship with social contact.

**Supplementary Information:**

The online version contains supplementary material available at 10.1186/s12302-021-00550-7.

## Background

Numerous studies have assessed the effects of environmental and socioeconomic variables on the spread of the COVID-19 pandemic. Most of them have addressed the influence meteorological variables have, although there are also quite a few that have considered the effects of air pollutants and socioeconomic variables. Those which assessed the effects of meteorological variables were the first to appear, specifically between the last week of March and the first week of April 2020. In other words, very close to COVID-19 being officially declared a global pandemic (11 March 2020) [1]. Later, there were those which evaluated the effects of air pollutants, the first of which appeared between the end of April and the first week of May 2020. Finally, the last ones to appear were those related to socioeconomic variables; the first of which was mid-May 2020.

The studies differ in their outcomes (new and cumulative cases, mortality, reproductive number, etc.), study populations (the world, countries, regions, cities), confounders as well as in the way of controlling for them, and in the modelling strategies adopted. However, with the exception of socioeconomic variables, several systematic reviews attempting to synthesize the evidence have already been published.

For instance, with regard to meteorological variables, Mecenas et al. carried out a bibliographic search until the end of March 2020 [2]. In reviewing 17 studies (most of them preprints), they found that warm wet climates seemed to reduce the spread of COVID-19. However, the role of temperature and humidity on the spread of the virus was very moderate, since these variables alone could not explain most of the variability in the disease’s transmission. Smit et al., in a systematic review carried out in July 2020 (that is, of studies that used data from the first wave), critically evaluated 42 articles published in scientific journals and 80 preprints [3]. They concluded that the evidence suggested that either there was no modulating effect of the summer weather conditions (i.e., high temperature and low humidity reduce the transmission rate of the virus) or, along the same lines as Mecenas et al., if it did exist, it was weak. Smit et al. also found similar results for other meteorological variables, such as ultraviolet radiation and wind speed [3]. McClymont and Hu discussed 23 articles with moderate or high ratings (out a total of 86 eligible peer-reviewed articles) published until October 1 (also contemplating only the first wave) [4], and found that temperature and humidity were associated with COVID-19 incidence. However, while the decrease in temperature was associated with increases in incidence, in the variations in humidity the results were mixed (positive and negative associations were found). They also found that wind speed and rainfall results were not consistent across studies [4].

In relation to air pollutants, Copat et al. carried out a systematic review of 15 studies (13 articles and 2 preprints) published between April 2020 and July 6th, 2020 [5]. They found a consistent association between some air pollutants (fine particles, PM_2.5_ with a diameter of 2.5 microns (μm) or less, and nitrogen dioxide, NO_2_, and with a less extent coarse particles, PM_10,_ with a diameter of 10 μm or less) and a higher incidence and mortality from COVID-19. They pointed out, however, that there were important limitations for any direct comparison of the results and that more studies were needed to strengthen scientific evidence. Malecki et al. carried out a systematic review of 19 studies, published through to October 31, 2020, that assessed the association of particulate matter (i.e., PM_10_ and PM_2.5_) pollution and the spread of SARS-CoV-2 [6]. They pointed out that although there were suggestions that particulate matter (PM) played a role in the spread of SARS-CoV-2, PM concentration alone cannot be effective in spreading the COVID-19 disease, and that other meteorological and environmental variables were also involved.

Until today (June 2021), no peer-reviewed systematic reviews have been published concerning the influence socioeconomic variables have on the spread of the pandemic. However, let us advance some of our results here by noting that in ecological studies the results were not conclusive. In some, especially those carried out in the United States, the areas with greater economic deprivation had a higher incidence and also a higher mortality. That said, in others no association was found, or deprivation was even found to be a protective factor. What was consistently observed was the fact that the higher the population density was, the greater incidence and mortality were. In individual studies, however, individuals with lower incomes or from more disadvantaged groups were at greater risk of hospitalization and death.

Nevertheless, all the reviews state that many of the studies have significant methodological limitations and errors that could bring their results into question. Our main objective here is to assess the methodological limitations in the studies that evaluated the effects environmental and socioeconomic variables have had on the spread of COVID-19. Furthermore, we discuss the results of those studies that were, in fact, able to control those very limitations.

## Methods

### Systematic review

The protocol for this review is registered in the Prospective Register of Systematic Reviews (PROSPERO 2020 CRD42020201540). In the review process, we followed the preferred reporting items for systematic reviews and meta-analysis (PRISMA) protocols [7]. The literature search, study selection, data extraction, and quality assessment were performed by each of us independently. In case of any discrepancy between us, we all reached an agreement on the final decision.

By combining the keyword ‘COVID-19’ with the keywords ‘temperature’, ‘(meteorological variables)’, ‘(air pollutants)’, ‘(environmental variables)’, and ‘(socioeconomic variables)’, through the Boolean connector ‘AND’ we conducted a search in the online databases PubMed, Web of Science and Scopus, up to December 31, 2020. We did not impose any language restrictions, nor did we contact any author for additional information.

**Fig. 1:**
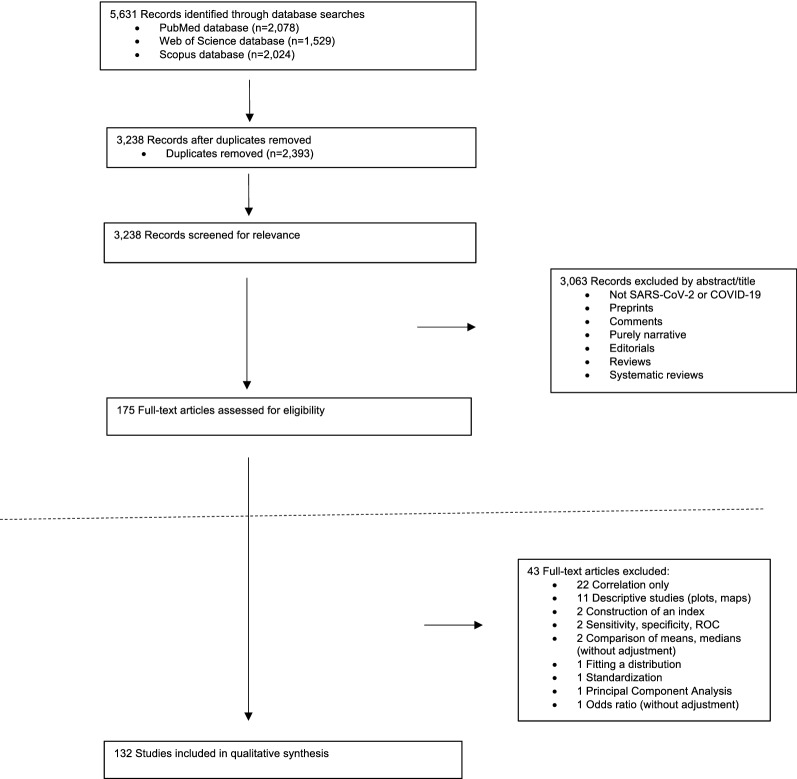
Flow-chart of the study selection process

All the articles retrieved underwent an initial title and abstract screening, where any duplicates were discarded, followed by a full-text screening for eligible abstracts. We made a first exclusion of those studies that did not deal with SARS-CoV-2 or COVID-19, preprints (non-peer-reviewed articles), comments, opinion or purely narrative papers, reviews and systematic literature reviews (Fig. 1). Among the eligible full-text articles, we made a second exclusion of those articles that were purely descriptive (including only plots or maps, etc.) and those that did not include any type of regression model (those that only included the analysis of correlations, for example).

We extracted the following data from the articles included in the qualitative analysis: first author, study population, study period, outcome, explanatory variables, covariates, the statistical method (including the model specification and the methods to control the confounding), and the study findings.

### Methodological limitations

The usual assessment tools for observational studies were not entirely suitable for assessing the risk of bias of the studies we reviewed. We preferred to adapt the tool proposed by Parmar et al. [8] who, in turn, adapted the Newcastle–Ottawa scale [9] and the RTI item bank [10]. Specifically, we used six domains: two from Parmar et al. [8]—confounding bias and measurement errors in the outcome and/or in the exposure variables; one based on the dimension ‘unobserved confounding’ in Saez et al. [11]—control of the spatial and/or the temporal dependence; and three that we added ex novo in this paper—control for the population, statistical model, and control of non-linearities.

In each study, each of the six domains were rated as: 1—low risk of bias, 2—moderate risk of bias, or 3—high risk of bias) (Table 1). For the overall rating of each study, we evaluated it as 'strong' (low risk of bias) if, at most, one of the six domains was rated as high risk of bias (i.e., a rating of 3), 'moderate' (moderate risk of bias) if up to two domains were rated as weak, or 'weak' (high risk of bias) if three or more domains were rated as high risk of bias. For the rating of both the domains and the studies, we rely on Parmar et al. [8].

**Table 1:** Bias assessment tool

Bias domain	Question to Consider	Indicator	Score (1: Low Risk of Bias; 2: Medium Risk; 3: High risk)
Confounding bias	Did the study analysis adjust potential confounders appropriately?	Confounders adjusted	1: Many confounders and unobserved confounding
			2: Some confounders (in particular mobility or socioeconomic variables) or unobserved confounders
			3: None or cannot tell
Control of the population	Did the study control for population?	Population, age structure of the population	1: Control and/or including population density
			2: Control only by including population density
			3: No control
Control of the spatial and/or temporal dependence	Did the study control the spatial and/or temporal extra variability?	Spatial and temporal dependence	1: Control
			2: Partial control (control of only one dependence, for example)
			3: No control
Control of non-linearities	Did the study control for non-linearities?	Non-linearities (parametric or non-parametric)	1: Control
			3: No control
Measurement errors	What was the heterogeneity of indicators used in the study?	Measurement errors in the explanatory variables (exposure variables and covariates)	1: Control
			2: Partial control (including lags, for example)
			3: No control
Statistical model	Did the study use appropriate statistical model?	Statistical model	1: Models for count data response variables
			2: Control of heteroscedasticity and rates as response variables
			3: Models with normally distributed errors and count data response variable; or no control of heteroscedasticity and rates as response variables
Overall Study Rating	Strong (low risk of bias): one domain, at most, was rated as 3		
	Moderate: up to 2 domains were rated as 3		
	Weak (high risk of bias): ≥ 3 domains were rated as 3		

Three of the six dimensions corresponded to the specification error known as omission of relevant variables: confounding bias, control of the population and control of the spatial and/or of the temporal dependence. This specification error leads to biased and inconsistent estimators (that is, the estimators biased even asymptotically, i.e., when the number of observations is very high) and, in addition, the variances of the estimators are also misleading [12]. In any case, the inference of those studies that do not control for this error is highly compromised.

### Confounding bias

None of the studies included all possible confounders, especially if the studies were ecological (as most of them were). However, as regards the spread of COVID-19, there is a confounder that, at a minimum, must be controlled for, namely, social contact.

The main route of transmission for COVID-19 is through the direct or indirect contact with an infected subject via the small droplets that occur when they cough or sneeze [13]. Thus, this contact must be controlled for in the models, even if indirectly. The control, although partial, can be carried out through mobility or, much more indirectly, through socioeconomic variables. In general, greater mobility implies greater levels of contact. Likewise, areas with high population densities are known to have greater social contact. Furthermore, some occupations present a greater risk, particularly those that were less able to switch to teleworking and, therefore, require greater mobility and the resulting higher level of social contact.

Unobserved confounding (i.e., residual confounding) including, for example, random effects that capture heterogeneity, should also be controlled for. In other words, unobserved variables specific to the unit of analysis (area or individual) that could influence the risk of, in this case, the spread of the COVID-19.

We scored this domain with a 3 if the confounding was not controlled for by any method, with a 2 if the observed confounding was controlled for with a moderate number of confounders (up to two maximum), in particular mobility or socioeconomic variables, or with a 1 if the observed confounding was controlled for with a large number of confounders (more than two) and/or unobserved confounding was also controlled for.

### Control of the population

Perhaps the main relevant variable that should not be omitted by any study is that of population at risk, either in the study area (in ecological studies) or in the area in which the subject resides (in individual studies). It is evident that both incidence and mortality, as well as other outcomes (hospitalizations, ICU admissions, etc.), depend both on the population of the area under study and on the age structure of that population.

Population control can be carried out in various ways: using rates, including the population or the expected value of the outcome in each area under study in the model as an offset, or controlling, as covariates, the size of the population or its structure (for example, percentage of population aged 65 years or more).

A control of the population can also be achieved by including population density (i.e., the number of people per unit of area, usually per square kilometre) as a covariate. However, it is possible that, in this case, control would only be partial. On one hand, an area with a higher population density does not always have more population than another, but it depends, logically, on its surface. On the other hand, population density could be capturing other socioeconomic variables.

This domain was scored with a 3 if the population was not controlled for by any method, a 2 if the population was controlled for by only including population density as a covariate, or a 1 if the population was controlled for, in addition to including the population density by other additional method.

### Control of the spatial and/or of the temporal dependence

Several studies analyze, as outcome, cumulative cases and cumulative deaths. Many others, however, use a temporal design. This is a design, where both the outcome and its possible explanatory variables, as well as the covariates, are measured in the form of time series. Time series are observed with a certain periodicity, usually regular (for example, daily) over a given period of time.

In this case there is temporal dependency. The outcome observations are not independent but are related, so their future behavior is predictable. In general, this dependence can be long or short term. A long-term dependency, or trend, could be defined as a movement or tendency in the data. As is known, in the case of COVID-19 there have been between two and four waves, depending on the country. That is, long-term swings have occurred. Periods in which the outcome values are persistently high, followed by others in which the values have been low. Short-term dependency, also called serial autocorrelation, refers to the relationship of the values of an outcome on, for example, a given day with the values of the previous days, especially with those of the preceding day.

Most studies use a spatial or spatio-temporal design. In other words, they observe the outcome in different geographical areas, and sometimes over time. When a spatial design is available, it is important to distinguish two sources of variation. In the first place, the most important source is usually the so-called 'spatial dependence' and is a consequence of the correlation of the spatial unit with neighboring spatial units, generally those that are geographically contiguous. In this way, the risks (for example, of transmission) of contiguous or nearby areas are more similar than the risks of spatially distant areas. Part of this dependency is not really a structural dependency but is mainly due to the existence of uncontrolled variables, that is, not included in the analysis. Meanwhile, the second source, the existence of spatially independent and unrelated variation called ‘spatial heterogeneity’, must be assumed. This is a consequence of the existence of unobserved variables without spatial structure that could influence risk [14].

The temporal and the spatial dependence must be controlled for, because, otherwise, in the best of cases, the variances of the estimators will be misleading (when the outcome is a continuous variable, normally distributed, and least squares methods are used for the inference) and in most cases, not only will the variances be biased, but the estimators will also be biased (when the outcome is not a continuous variable, not normally distributed, and least squares methods cannot be used) [12].

In some studies, the control of temporal or spatial dependence is not applicable. Thus, in studies with a time series design but in which a very short period of time is analyzed, it does not make sense to control for temporal dependence. Likewise, in those studies with a spatial (or spatio-temporal) design but that analyze very spatially distant territories (for example, several countries in the world) it does not make sense to control for the spatial dependence.

We scored this domain with a 3 when neither temporal nor spatial dependency was controlled and should have been; a 2 when the control was partial, controlling only one dependency and not controlling the other; and a 1 when they were controlled.

### Control of non-linearities

**Fig. 2:**
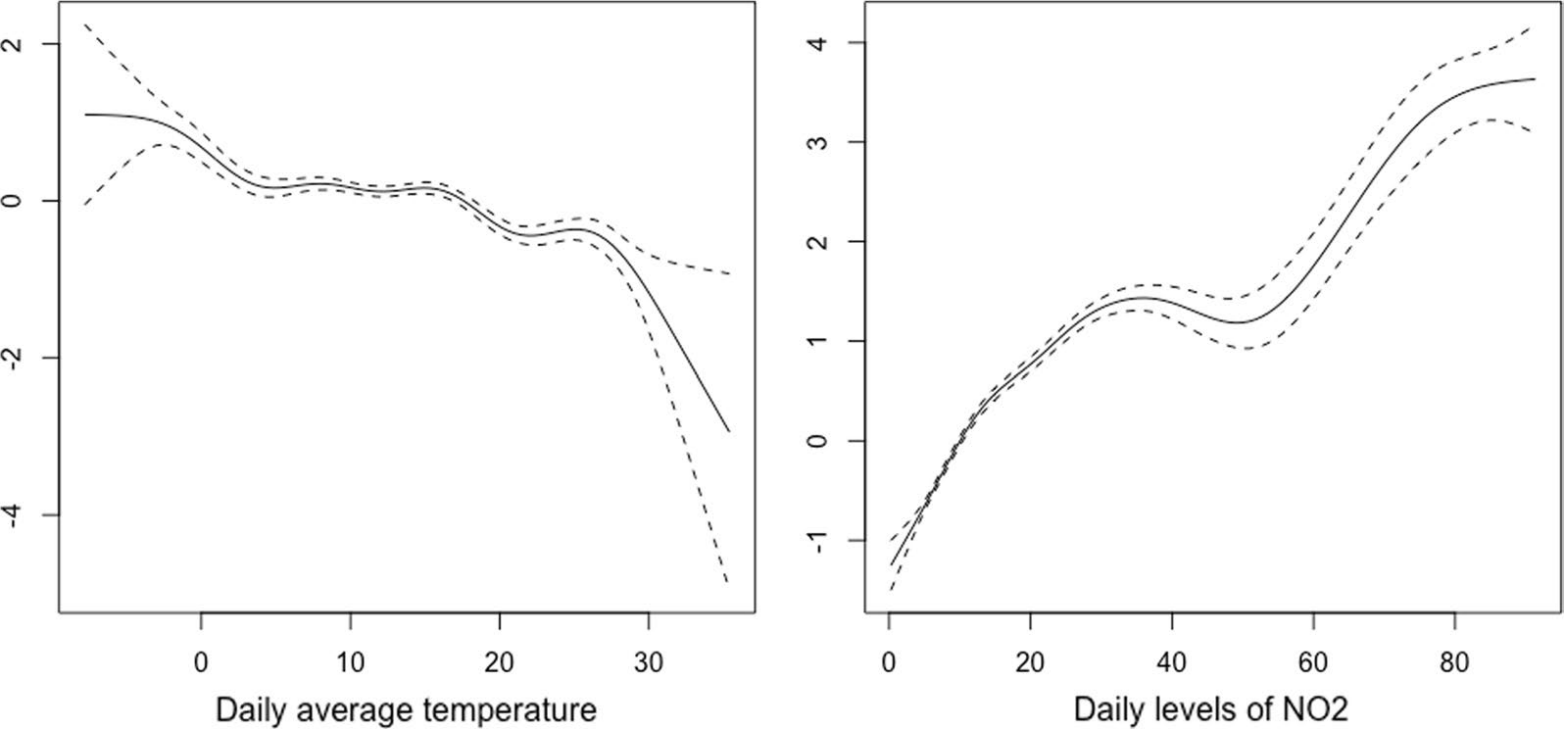
Smoothed curves for the relationships between daily temperature and daily levels of nitrogen dioxide and the number of daily cases of COVID-19. Spain, January 1, 2020 to April 14, 2021. The data were obtained from: [16] . Environmental data [81, 82]

Along with the omission of relevant variables, the error in the functional form constitutes the most important specification error. The relationships between environmental variables and COVID-19 outcomes are not usually linear. Thus, for example, in Fig. 2, we show the smoothed curves for the relationship between the daily temperature and the daily levels of nitrogen dioxide (NO_2_) and the daily number of cases for Spain in the period between January 1, 2020 and April 14, 2021. Specifically, we draw the estimated curves in a generalized additive model in which we use smoothing splines with a quasi-likelihood Poisson link, i.e., taking into account over-dispersion.

As can be seen, in none of the cases was the relationship linear. These non-linearities must be controlled in the models, because, otherwise, as when relevant variables are omitted, the estimators will be inconsistent and their variances misleading.

We scored this dimension with a 3 if non-linearities were not controlled for (again, when applicable) or a 1 if they had been controlled.

### Measurement errors

Measurement errors (also known as misclassification) can occur in both the response variable and in the exposure variables.

The definition of the response variable can vary in space and time, even within the same country, leading to differential misclassification. In Spain, for example, the Catalan government, on the one hand, defined a death from COVID-19 as being a positive result on some test (PCR or fast test) or symptoms presented at some point which a health professional subsequently classified as a possible case, but the individual did not have a diagnostic test with a positive result [15], whereas on the other hand, the Spanish government, defined a death from COVID-19 as being someone who presented a positive PCR result [16], thus providing significantly lower figures. This misclassification continued until May 21, 2020, when the Government of Spain adopted the same definition as the Government of Catalonia [17].

However, the measurement errors in the response variable are not attributable to the investigators, although they should certainly discuss them if appropriate. Furthermore, fortunately, when measurement errors occur in the dependent variable, the estimators remain consistent, although they are not efficient [12], that is, not very precise, thus leading to wider confidence intervals than if there had been no measurement errors.

There is, however, an important problem if measurement errors occur in the explanatory variables (exposure or covariates). If the explanatory variables are measured with error, the estimators will be inconsistent [12].

Even in studies at the individual level, the exposure variables and, obviously, the contextual variables (for example, the socioeconomic ones) are not observed at the individual level, but are aggregated at the level of the area under study. Nevertheless, not all residents in the area under study are actually exposed to the same mean values of the explanatory variables, which leads to a measurement error. If the misclassification is non-differential (over time and over space within the area under study) and, furthermore, if the between-area variability of the variable measured with error is much greater than the within-area variability of such variable, that is, that the area under study is not very heterogeneous (for example, because it is a small area), then the effect of the measurement error on the estimator consistency may be negligible [18]. This is what happens in the case of contextual socioeconomic variables as long as the area under study is not very large.

In the exposure variables (both air pollutants and meteorological variables), however, there is differential misclassification, because the exposure exhibits spatial variation across the area under study. If the spatial structure (i.e., spatial dependence) of the data is ignored, the estimators will be biased and inconsistent [19]. Many studies use the measurements observed in the area under study to estimate, by means of point estimators, exposure levels for that entire area. The estimators most widely used are the arithmetic mean of the values of the exposure, observed in several monitoring or meteorological stations in the area, and sometimes the inverse-distance weighted average of these values.

This measurement error in the exposure variables must be controlled for, either explicitly incorporating the spatial dependence, in the ecological studies, or by correcting the misalignment between the locations of the observation points of the exposure variables and that locations of the individuals, in the studies at the individual level.

In studies with an ecological spatial design, the 'modifiable areal unit problem' (MAUP) occurs [20]. The MAUP is a consequence either because areas of different sizes are added (scale effect) and/or because of the way the area is divided (zoning effect) [21]. In either case, it is a potential source of bias. For example, Wang and Di found that the association between nitrogen dioxide (NO_2_) and COVID-19 deaths varies when the data is aggregated at different levels: a risk factor when the area is smaller (aggregation of districts and cities) and a protective factor at the province level [22]. Similarly, we also found a positive association between NO_2_ and deaths as a consequence of COVID-19 at the level of a county-like area [17] and no association at a lower level of aggregation [23].

When using a temporal design, the ‘modifiable temporal unit problem’ (MTUP) [24] also occurs, whereby the results depend on the way data are temporally aggregated [21]. Furthermore, in this type of design, temporal misalignment can occur. In other words, the relationship between exposure and the occurrence of COVID-19 outcome is not contemporary, but rather is distributed over time as a consequence of the incubation period of COVID-19 and due to the diagnostic delays of the outcome. This temporal misalignment must be controlled by including lags, for example.

We scored this dimension with a 3 if measurement errors in the exposure variables are not controlled at all, a 2 if they are only partially controlled (not including lags, for example) or the areas under study are very large (countries, for example) and a 1 if they have been controlled for.

### Statistical model

Many of the studies, even though the response variable is a count data, used regression models with normally distributed errors (linear regression models, generalized linear and additive models with Gaussian link, etc.). Using this type of models leads to biased results, unless the number of counts is very large. However, this was not the case in most studies.

Some studies did not model the counts but rather the rates, dividing the dependent variable by the size of the population. However, since the numerator, being a count data, is actually distributed following a Poisson distribution, the variance is proportional to the mean, so it is not constant, leading to heteroscedasticity (i.e., overdispersion). This must be controlled for, otherwise, the variances of the estimators are misleading.

**Fig. 3:**
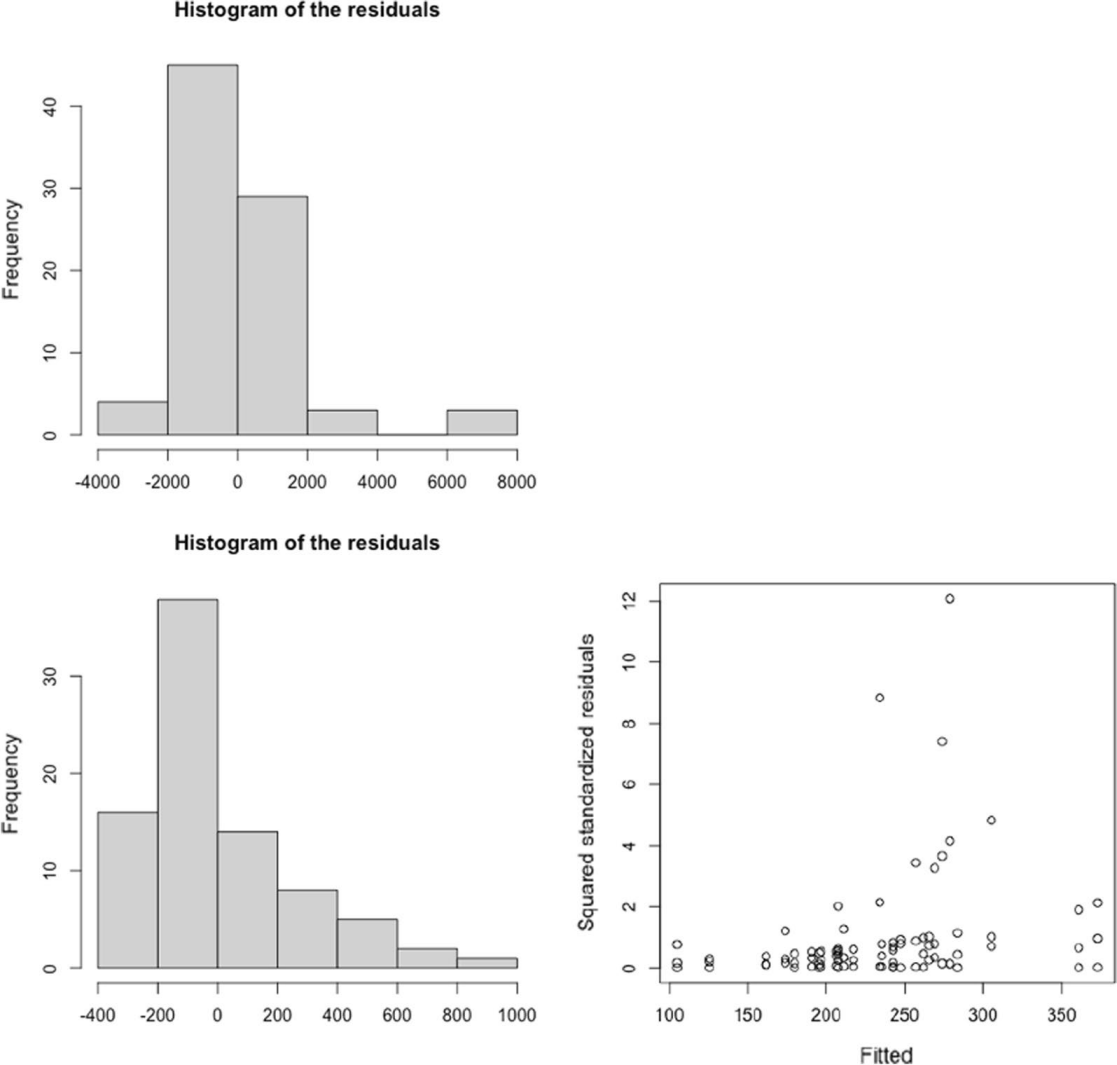
Residual analysis of the linear regression models relating the transmission of SARS-CoV-2 infection and long-term exposure to NO_2_ in the provinces of three regions of Northern Italy (Lombardia, Venetto and Emilia Romagna), between March 8 and April 5, 2020. **a** Response variable: new daily SARS-CoV-2 positive cases. **b** Response variable: new daily SARS-CoV-2 positive cases per 100,000 habs. The data were obtained from: [25]

To illustrate the effects on the results of erroneously using a regression model with normally distributed errors, we used the data in Filippini et al. [25]. Their objective was to investigate the link between the transmission of SARS-CoV-2 infection and long-term exposure to NO_2_ in the provinces of three regions of Northern Italy (Lombardia, Venetto and Emilia Romagna), between March 8 and April 5, 2020 (*n* = 84). Using their data, we first estimated a linear regression model including, as a dependent variable, the number of new daily SARS-CoV-2 positive cases (count data variable). We found that long-term NO_2_ levels to which the inhabitants of the provinces of the Italian regions studied had been exposed to be positively associated with the total number of cases that occurred in the period considered. Specifically, for every 1 μg/m^3^ increase in the NO_2_ levels, the number of cases increased by 18.478 for the entire period (95% confidence interval, 95% CI 10.285–27.210). However, the residuals of the model were not normally distributed (Fig. 3). We then modelled the rates (cases per 100,000 inhabitants) using a linear regression model, although we did not control for heteroscedasticity. For every 1 μg/m^3^ increase in NO_2_, the number of cases increased by 1.207 cases per 100,000 inhabitants (95% CI 0.050–2.364). However, the residuals presented a clear heteroscedasticity behavior (the scatter plot of the residuals against the adjusted values did not present a constant dispersion, i.e., variance), and furthermore, they were not normally distributed (Fig. 3). When we estimated a generalized Poisson model, in which we took into account the over-dispersion, and in which we included the population size as an offset, we could not reject the null hypothesis that the parameter associated with the long-term exposure of NO_2_ was equal to zero (95% CI: − 0.004, 0.001).

We scored this dimension with a 3 when the outcome was a count data and regression models with normally distributed error were used. We also scored a 3 when rates were modelled but heteroscedasticity was not controlled for. Meanwhile, we scored a 2 if rates were modelled and heteroscedasticity was controlled, and a 1 if models for count data response variables were used (Poisson regression, negative binomial regression, etc.).

## Results

### Systematic review

Figure 1 shows a flowchart of the review process. Of the 5631 abstracts initially identified, and after excluding duplicates, we were left with 3238 studies. From these we excluded 3063 studies that did not refer to SARS-CoV-2 or COVID-19, preprints, comments, those purely narrative studies, editorials and reviews and systematic reviews, thus leaving us with 175 eligible studies. As we said, we excluded 43 studies that were purely descriptive and those that did not include any type of regression model (Additional file 1: Table S1). In the end we were left with 132 studies with which to carry out the qualitative synthesis (Additional file 1: Tables S2 and S3).

Of the 132 studies, 92 referred to meteorological variables, 40 to socioeconomic variables and 34 to air pollutants. Seventy-one of the studies referred only to meteorological variables, 21 only to socioeconomic variables and 16 only to air pollutants. Of the 92 studies that referred to meteorological variables, 16 also considered air pollutants, 14 meteorological variables and socioeconomic variables. Four of the studies referring to air pollutants also referred to socioeconomic variables but not to meteorological variables. Nine referred to meteorological variables and socioeconomic variables but not to air pollutants. Finally, five studies considered meteorological variables, air pollutants and socioeconomic variables (Additional file 1: Figure S1).

Of the 132 studies finally selected, 124 used an ecological design and nine an individual design. Most ecological studies considered different regions (states, regions, provinces, counties, cities, etc.) within the same country as study populations (71 studies). This is followed by those that considered countries or cities in the world (34 studies) and, finally, those that considered individual cities or smaller areas (19 studies). Seven of the eight studies with an individual design, analyzed the influence of socioeconomic variables, while only two considered socioeconomic variables and air pollutants.

Most of the studies (129 out of 132) analyzed data referring up to August 1, 2020 (i.e., only considering the first wave). In fact, only three consider the first two waves of the pandemic.

### Methodological limitations

Table 2 shows the evaluation of the studies included in the qualitative synthesis. Of the 132 eligible studies, we evaluated 63.64% (84 of 132) as weak (high risk of bias), 19.70% (26 of 132) as moderate (moderate risk of bias) and 16.67% (22 of 132) as strong (low risk of bias). Only four studies did not have any dimension scored with a 3 (high risk of bias) [17, 26–28].

**Table 2:** Evaluation of bias for the studies in the systematic review

Manuscript	Confounding bias	Control of the population	Control of the spatial and/or temporal dependence		Control of non-linearities	Measurement errors	Statistical model	Overall rating
Aabed	3	2	3		1	3	1	***3***
Adekunle	3	3	1		1	2	3	***3***
Adhikari	2	3	2		3	2	1	*2*
Ahmadi	1	2	3		3	3	3	***3***
Azar	1	2	3		NA	2	1	**1**
Azuma	1	1	2		3	3	3	***3***
Behnood	3	2	3		1	3	3	***3***
Briz-Redón	1	1	1		1	3	3	*2*
Byass	3	1	3		3	3	1	***3***
Carleton	3	1	2		1	3	3	***3***
Chadeau-Hyam	1	3	2		3	2	1	*2*
Chakrabarty	3	3	3		1	3	1	***3***
Chakraborty	1	1	3		2	3	1	*2*
Chaudhry	1	2	3		NA	3	1	*2*
Chien	1	1	1		1	3	1	**1**
Coccia a	3	2	3		3	3	3	***3***
Coccia b	1	2	3		3	2	3	***3***
Coker	1	1	2		3	1	1	**1**
Das a	3	1	3		NA	3	1	***3***
Das b	1	3	3		NA	3	1	***3***
Demongeot	3	3	3		3	3	3	***3***
DiMaggio	1	1	1		NA	2	1	**1**
Dogan	3	3	3		3	2	3	***3***
Drefahl	1	3	3		NA	2	1	*2*
Falcão Sobral	2	2	3		3	3	3	***3***
Fattorini	3	3	3		3	3	3	***3***
Fazzini	3	3	3		3	3	3	***3***
Fiasca	3	2	3		3	3	3	***3***
Filippini	1	1	3		1	3	3	***3***
Fu	2	2	2		3	2	1	*2*
Guasp	2	2	3		3	3	3	***3***
Guo C	1	1	3		1	2	1	**1**
Guo XJ	3	3	3		3	3	3	***3***
Gupta	3	3	NA		3	3	3	***3***
Han	1	3	1		3	2	3	***3***
He	3	3	1		1	3	1	***3***
Hoang a	2	3	3		1	3	3	***3***
Hoang b	2	3	3		1	1	3	***3***
Hutter	2	2	3		3	3	1	***3***
Iqbal MM	3	3	NA		3	3	3	***3***
Iqbal N	2	3	3		1	3	3	***3***
Isaia	1	1	3		3	3	3	***3***
Islam ART	3	3	3		1	2	1	***3***
Islam N	2	1	3		2	3	1	*2*
Jamshidi	1	2	3		3	3	3	***3***
Jiang	3	3	3		3	3	1	***3***
Jüni	1	2	NA		3	2	2	**1**
Kaiser	1	2	NA		NA	3	3	*2*
Khan	3	1	3		1	3	3	***3***
Kodera	3	2	3		3	3	3	***3***
Kubota	1	1	2		3	3	3	***3***
Lamb	1	2	2		NA	3	3	*2*
Lau	2	3	3		NA	2	3	***3***
Lhada	2	3	3		3	3	3	***3***
Li AY	1	2	3		3	3	3	***3***
Li H	3	3	3		3	3	3	***3***
Li X	2	3	3		NA	2	3	***3***
Liang	1	1	1		3	2	1	**1**
Lin	2	2	3		3	3	3	***3***
Liu	2	3	2		1	2	1	**1**
López-Feldman	1	1	3		3	2	1	*2*
Luo	1	1	3		1	3	2	*2*
Ma	3	1	2		1	3	1	*2*
Madhav	1	1	3		NA	2	1	**1**
Malki	3	3	3		1	3	3	***3***
Mandal	3	1	NA		3	3	3	***3***
Marciel de Souza	3	1	1		NA	3	1	*2*
Martorell-Marugan	3	1	3		3	3	3	***3***
Medeiros-Figuereido	1	2	3		3	3	3	***3***
Meo	1	3	NA		3	3	3	***3***
Meraj	3	3	3		3	3	3	***3***
Meyer	1	1	1		3	2	1	**1**
Muñoz-Cacho	3	2	3		3	3	3	***3***
Notari	3	3	1		1	3	3	***3***
Ozyigit	1	3	3		3	3	3	***3***
Paez	2	2	2		3	3	3	***3***
Pan	3	3	3		3	3	3	***3***
Pequeno	1	2	3		3	2	1	*2*
Perone	1	1	3		3	3	3	***3***
Pirouz	2	3	3		3	2	3	***3***
Plümper	2	2	2		NA	2	3	**1**
Poirier	2	3	3		3	3	3	***3***
Pozzer	NA	2	NA		1	1	1	**1**
Pramanik a	3	3	3		1	3	3	***3***
Pramanik b	3	3	3		1	3	3	***3***
Prata	2	1	1		1	3	1	**1**
Price-Haywood	1	2	3		NA	2	1	**1**
Qi	2	1	3		1	3	1	*2*
Rafael	3	2	3		NA	2	3	***3***
Rahman	1	1	3		3	3	3	***3***
Rashed	3	3	3		3	3	3	***3***
Rehman	1	1	3		3	3	1	***3***
Richmond	1	1	3		NA	2	3	*2*
Rodríguez-Villamizar	1	1	2		3	3	1	*2*
Rozenfeld	1	3	3		NA	2	1	*2*
Rubin	1	3	3		1	2	1	*2*
Runkle	1	2	2		3	3	1	*2*
Saez	1	1	1		1	2	1	**1**
Sajadi	1	3	NA		3	3	3	***3***
Sánchez-Lorenzo	3	3	NA		3	3	3	***3***
Sannigrahi	1	2	3		NA	2	3	*2*
Sarkodie	1	3	1		3	2	3	***3***
Scarpone	1	1	1		1	2	3	**1**
Sehra	1	1	3		3	3	1	***3***
Shahzad F	3	3	3		3	3	3	***3***
Shahzad K	3	3	3		3	3	3	***3***
Shao	2	3	NA		3	3	1	***3***
Shi	3	1	2		1	2	1	**1**
Stieb	1	1	2		3	3	1	*2*
Su	1	3	NA		3	3	3	***3***
Sun	1	1	1		3	3	3	***3***
Tagaki a	2	1	3		3	3	3	***3***
Tagaki b	3	3	3		3	3	3	***3***
To	3	1	2		3	3	3	***3***
Tobías	2	1	2		3	2	1	**1**
Tzampoglou	1	1	3		3	3	3	***3***
Ujiie	1	1	3		3	3	1	***3***
Wang Q	1	1	2		1	3	1	**1**
Wang Y	3	3	3		1	3	3	***3***
Ward a	3	1	3		1	3	1	***3***
Ward b	3	1	3		1	3	1	***3***
Wu X	1	1	2		1	2	1	**1**
Wu Y	2	2	NA		1	3	3	*2*
Xie J	2	1	2		1	2	1	**1**
Xie Z	1	3	1		3	3	3	***3***
Xu	3	1	2		3	2	1	*2*
Yao a	1	3	3		3	3	2	***3***
Yao b	1	3	3		3	2	3	***3***
You	1	2	1		NA	3	3	*2*
Zakeri	1	2	3		NA	2	1	**1**
Zhang	2	3	3		1	3	3	***3***
Zhu L	3	3	2		1	2	3	***3***
Zhu Y	3	3	NA		3	3	2	***3***
Dimensions				Overall				
Number of 3 s	47	53	80		73	90	77	85
Number of 2 s	26	32	23		2	40	4	26
Number of 1 s	60	48	30		58	3	52	22

Bold: Strong (rate 1); Italic: Moderate (rate 2); Bolditalic: Weak (rate 3)

In decreasing order of the studies that considered socioeconomic variables, 62.50% (25 of 40 studies) were evaluated as moderate (15 studies, 37.50%) or strong (10, 25.00%). Of the 34 which considered air pollutants, 41.18% (14 studies) were evaluated as moderate (9 studies, 26.47%) or strong (5 studies, 14.71%). Finally, of the 92 studies that considered meteorological variables, 25.00% (23 studies) were evaluated as moderate (11 studies, 11.96%) or strong (12 studies, 13.04%).

However, in the case of studies that consider socioeconomic variables, it should be noted that the high risk of bias could be underestimated. As is known, socioeconomic variables are contextual variables measured at an ecological level in a geographic area and invariant over time. Their influence on COVID-19, if any, is highly unlikely to be non-linear. Consequently, in many cases this dimension was not evaluated.

The dimension in which we evaluated more studies with a high risk of bias was that of measurement errors (90 of 132 studies, 68.19%), followed by the control of the spatial and temporal dependence dimension (80 studies, 60.61%) and of the statistical model (77 studies, 58.33%) and control of non-linearities (73 studies, 55.30%) dimensions. The dimensions with fewer studies with a high risk of bias were confounding bias (47 studies, 35.61%) and control of the population (53 studies, 40.15%).

### Findings from studies assessed as moderate or strong

In relation to the studies that considered meteorological variables, the ones that we evaluated as moderate or strong [28–48] have not consistently found an attenuating effect of meteorological variables. That is, they have not found that high temperature and low humidity were associated with lower incidence or mortality from COVID-19. In seven of 22 studies, temperature was either positively associated or not statistically associated with incidence [27, 30, 34, 35, 44], transmission (reproductive number) [46] and mortality [28] (four out of 11 studies were assessed as strong and another three out of 11 studies assessed as moderate). Among the studies that found an attenuating effect, five (three evaluated as strong [31, 38, 40] and one as moderate [41]) did not include lags and, therefore, assumed that the effect of the meteorological variables was contemporaneous. The studies that did include lags were evaluated with high risk of bias in some dimension. In particular, control of non-linearities [32, 39, 42, 45, 48], confounding bias [37, 43, 48], and measurement errors [37, 42, 47], followed by control of population [29, 36] and control of spatial and/or temporal dependence [33, 39]. Interestingly, Xie et al. [27], whose units of analysis were 122 Chinese cities, (a study that we evaluated as strong and did not have any dimension evaluated as high risk), points out that there is no evidence supporting that case counts of COVID-19 could decline when the weather becomes warmer.

There was very little evidence in relation to other meteorological variables such as wind speed (only two strong [33, 49] and one moderate [34] study analyzed it and found a negative association between wind speed and incidence); cloud percentage [29] or solar radiation [42] (both evaluated as moderate and with contradictory results: higher percentage of cloud was associated with higher incidence, while no association was found with solar radiation); or precipitation (considered in only one strong study that found a significant negative association with incidence [31]).

Greater consistency was found in the association between greater exposure to levels of air pollution, especially long-term exposure, and an increase in COVID-19 outcomes, both in ecological [17, 28, 29, 44, 48–51, 54–56] and individual studies [52, 53]. The areas that were most exposed to air pollution were those with the highest incidence (new daily cases, new positive tests, and cumulative cases) [17, 29, 44, 48, 49, 53, 54] and the highest mortality [17, 28, 29, 49–52, 55, 56] from COVID-19. This result occurs, above all, for fine particles, PM_2.5_ [28, 44, 50–56], but also for ozone, O_3_ [29, 49, 50], coarse particles, PM_10_ [17, 50], nitrogen dioxide, NO_2_ [17, 50], benzene [55] and for an air quality index [48]. In Saez et al. [17] (which we evaluated as strong) as in Adhikari et al. [29] and Rodríguez-Villamizar et al. [56] (these last two evaluated as moderate), some of the pollutants were not found to be associated with mortality (PM_10_ in Saez et al., O_3_ in Adhikari et al., PM_2.5_ in Rodríguez-Villamizar et al.).

In relation to studies that considered socioeconomic variables, as we said, we must distinguish between the findings of ecological [17, 28, 54–66] and individual studies [53, 67–72]. In the ecological studies, there was no consistent association between socioeconomic contextual variables and COVID-19 outcomes. In just over half of the studies, the socioeconomic variables were risk factors and in the rest they were either protective factors or no statistically significant association was found. Even in some studies, such as Saez et al. [17] or Wu X et al. [28] (both of which we evaluated as strong and did not have any dimension evaluated with high risk of bias), apparently contradictory results were found. Thus, in Saez et al. [17], whose unit of analysis were small areas (counties and health zones, some made up of census tracts, others by municipalities) in Catalonia, Spain, the higher the percentage of poor housing in the small area and the more economically deprived the area was, the greater the risk of a positive result and death. Conversely, the higher the unemployment rate and the percentage of foreigners in the small area, the lower the risk of a positive result and death. In Wu et al. [28], whose units of analysis were US counties, while percent of the adult population with less than high school education and percent of Black residents, both in the county, were found to be positively associated with the number of deaths in the county, the median household income, the percentage of owner-occupied housing and, marginally, the median house value were also found positively associated. Meanwhile, others, such as the percentage of people in the county in poverty, were not found to be statistically significant associated.

More consistency has been found in relation to population density. In the areas with a higher population density, there was a higher incidence, a higher number of positives, a higher transmission (measured by the reproductive number) and a higher number of deaths than in others less densely populated areas. In Wu et al. [28], however, the higher the population density, the lower the risk of mortality (although statistical significance only occurs in the fourth quintile).

Of the seven individual studies that we evaluated as moderate or strong, five found an association between both individual socioeconomic status (income, non-white ethnicity—especially Blacks-, lower educational attainment, being an immigrant from a low- or middle-income country) and contextual (income of the area, where the subject resided, residing in a neighborhood with financial insecurity) and various COVID-19 outcomes (positive tests, hospital admissions and deaths). We did, however, find one exception. In Price-Haywood et al. (a study that we evaluated as strong), whose study population was the Ochsner Health facility in New Orleans, Louisiana, USA, Black race was not associated with higher in-hospital mortality than white race, after adjustment for differences in sociodemographic and clinical characteristics on admission [70].

## Discussion

Our results, both with regard to the methodological limitations that we found in the review and the results of the studies that control them, were similar to those of other reviews. Regarding the methodological limitations, we will refer, in order of publication, to two reviews (not systematic): one that considered air pollutants [73] and the other meteorological variables [74]. Villeneuve and Goldberg review six studies on COVID-19 (only two were peer-reviewed) and two on SARS, published up to May 2020 [74]. Hunter Kerr et al. review 43 studies (23 of them peer-reviewed), published in 2020 [74]. Both reviews found, as we did, that all studies have methodological limitations in one way or another. Almost all the methodological limitations that we have pointed out here were also considered in these two reviews. There are, however, some differences. Hunter Kerr et al. did not consider choosing a statistical model with normally distributed errors [74] as a limitation. Villeneuve and Goldberg, for their part, did not consider the error of the functional form (i.e., control of non-linearities), at least directly, inasmuch as they do so indirectly by pointing out, as a limitation, the inadequate evaluation of effect modification [73]. In contrast, Villeneuve and Goldberg point out, as the most important error, possible cross-level bias in ecological studies.

Regarding the influence of environmental variables (meteorological and air pollutants) in COVID-19 outcomes, the findings of the studies evaluated as moderate or strong in our review, coincided with the findings of the other reviews (both systematic and non-systematic).

We cannot conclude that there was an attenuating effect of weather conditions on the spread of the COVID-19 pandemic. In addition to the fact that, as mentioned, we did not find a systematic behaviour in the reviewed studies, so the attenuation shown by some of them could actually be a consequence of an inadequate adjustment. Thus, on the one hand, the study period of all the studies reviewed by the systematic reviews of Mecenas et al. [2], Smit et al. [3] and McClymont and Hu [4] as well as by the Hunter Kerr et al.’s review [74], corresponded to the first wave. The same occurs with most of the studies in our review (all except three). However, with a single exception [45], none of the studies controlled for non-pharmaceutical interventions either as containment or suppression strategies undertaken in that period. Thus, in this case, the reduction in the spread of the pandemic as temperature increased and humidity decreased, could have been confounded by the effects of lockdowns and other restrictions. Although Tobías and Molina [45] controlled for the effects of lockdown (and also those of seasonality as a consequence of weekends), they did not adjust for other confounders. Consequently, and perhaps for this reason, they found a significant effect only in the contemporary association (the same day) between an increase in temperature and a reduction in the incidence rate. We believe that, if they exist, the effect of meteorological variables on the spread of COVID-19 would be indirect. In the spring–summer of 2020, better weather conditions (higher temperature, lower relative humidity, lower wind speed, etc.) and a relaxation of restrictions, led to greater mobility and, therefore, greater social contact that, in turn, led to an increase in transmission and, consequently, in incidence. This was what happened, for example, in Spain during the second wave (which began in August 2020) [23].

The results of all reviews, including ours, suggest that there is an association between exposure to air pollutants (particularly in the long term but also in the short term) and COVID-19 outcomes. In fact, two hypotheses have been suggested that would explain this association. First, some studies have proposed that air particulate matter can operate as a virus carrier, promoting the spread of the SARS-CoV-2 [74–76]. It should be noted, however, that these studies were either not eligible as they used only correlation analysis to test their hypothesis [75] or they were eligible but were assessed as a high risk of bias [76].

A second hypothesis has been proposed which suggests there could be potential biological mechanisms that may explain the association between air pollutants and respiratory viral infections. According to this, the effects of exposure to air pollutants would occur not so much on transmission or incidence but on the worsening of the disease (hospitalization, ICU admissions, mortality). Exposure exacerbates the severity of COVID-19 infection symptoms and worsens the prognosis of COVID-19 patients [73]. In this sense, Wu X et al. [28] argue that long-term exposure to PM_2.5_ could cause alveolar angiotensin-converting enzyme 2 (ACE-2) receptor overexpression and impairs host defences [77]. This could cause a more severe form of COVID-19 in ACE-2—depleted lungs, increasing the likelihood of poor outcomes, including death [78]. We, however, believe that air pollutants have actually been surrogates of other variables, such as the mobility of residents and several socioeconomic conditions (high population density, poor housing, use of public transport, occupations in which it is not possible to telecommute, etc.) that facilitate social contact [17]. In fact, Dey and Dominici, in a very recent editorial commenting on the study by Wu et al. [28], and of which Dominici is a co-author, point out that the health risks of some racial subgroups are spiraling as they have higher levels of exposure to air pollutants, hence being more susceptible to mortality from COVID-19 [79]. We do not deny that exposure to air pollutants had an independent effect on, above all, the worsening of the disease among those diagnosed with COVID-19. However, we are convinced that this effect cannot be observed using an ecological design.

As we noted, we have found a consistency in the effects of socioeconomic variables on COVID-19 outcomes only in individual studies and in indicators also at the individual level (ethnicity—particularly being Black—education, etc.). We believe that the effect, if it exists, would be indirect. Poorer socioeconomic conditions would be associated, on the one hand, with greater social contact, which would affect the transmission of the virus and the incidence of COVID-19 and, on the other, with a greater number of comorbidities and greater difficulties in accessing health care which would affect a poorer prognosis of the disease. Furthermore, poorer socio-economic conditions could be related both to a differential exposure to air pollution and to a differential susceptibility to its effects (i.e., modification of the effect) [80].

In short, a large part of the methodological problems that we have encountered and, therefore, of the uncertainty in the findings, are the consequence of using an ecological design. In this sense, we could not agree more with Hunter Kerr et al. [74], who recommend, as an epidemiological design, a longitudinal study with individual-level data, in which those diagnosed with COVID-19 would be followed through time.

Our study may have three limitations. First, some studies published during 2020 may have escaped us. That said, this is unlikely, since, as of January 2021, we have been regularly reviewing PubMed and periodically reviewing the other databases. Nevertheless, it is not impossible that a study may have eluded us. Second, both the information extraction and the quality control we carried out could have some subjectivity. We have tried to minimize this as much as possible.

Finally, as we noted, the rating of both the domains and the studies are based on Parmar et al. [8], with the only difference being that in Parmar et al., an overall rating of strong was given if none of its domains was rated as weak. In our case, this assignment seemed too restrictive. In fact, applying this criterion would imply that only one of the studies could be rated as strong. In our case, we observed some biases that were not contemplated in Parmar et al., such as the lack of control of the population and of the spatial and/or temporal dependence, the non-control of non-linearity and the inappropriate use of statistical models. In our case, the probability that at least one of these biases occurred was very high. In any case, we admit that there could be some degree of arbitrariness in the assignment of the overall rating to one category or another.

## Conclusions

All the studies we reviewed have methodological limitations to a greater or lesser extent. Even those that we have evaluated as strong (16.67% of the studies reviewed) and, among them, those in which we did not evaluate any dimension as having a high risk of bias (4 studies), have the limitation of using an ecological epidemiological design or, in any case, either of measuring the exposure in an ecological way (exposure misclassification). These limitations prevent conclusions about the effects of environmental (meteorological and air pollutants) and socioeconomic variables on COVID-19 outcomes being drawn. However, we dare to argue that the effects of these variables, if they exist, would be indirect, based on their relationship with social contact. In any case, the estimation of these independent effects requires the use of an individual design and the control of the methodological limitations explained in this work. Among them, an estimate of individual exposure free of biases (non-differential misclassification, non-existence of spatial–temporal misalignment, etc.).

## Supplementary Information

**Additional file 1: Table S1**. List of studies excluded. **Table S2**. Studies included in the qualitative synthesis **Table S3**. List of studies included in the qualitative synthesis. **Figure S1.** Number of studies by type of explanatory variable analyzed

## Data Availability

All the studies, as well as the code to make the figures, can be requested from the corresponding author (marc.saez@udg.edu).

## Abbreviations

COVID-19: Novel coronavirus disease
PM_2.5_: Fine particles with a diameter of 2.5 microns (μm) or less
NO_2_: Nitrogen dioxide
PM_10_: Coarse particles with a diameter of 10 μm or less
SARS-CoV-2: Severe acute respiratory syndrome coronavirus 2
PM: Particulate matter
PROSPERO: Prospective Register of Systematic Reviews
PRISMA: Preferred reporting items for systematic reviews and meta-analysis
RTI: Item Bank for Assessment of Risk of Bias and Precision for Observational Studies of Interventions or Exposures
PCR: Polymerase chain reaction
MAUP: Modifiable areal unit problem
MTUP: Modifiable temporal unit problem
