# Supplementary material for: Methodological limitations in studies assessing the effects of environmental and socioeconomic variables on the spread of COVID-19: a systematic review
Source: Environ Sci Eur. 2021 Sep 10;33(1):108. doi: 10.1186/s12302-021-00550-7 (PMC8432444; doi:10.1186/s12302-021-00550-7)

| **Section and Topic** | **Item #** | **Checklist item** | **Location where item is reported** |
| --- | --- | --- | --- |
| **TITLE** | | |  |
| Title | 1 | Identify the report as a systematic review. | Title page |
| **ABSTRACT** | | |  |
| Abstract | 2 | See the PRISMA 2020 for Abstracts checklist. | 1 |
| **INTRODUCTION** | | |  |
| Rationale | 3 | Describe the rationale for the review in the context of existing knowledge. | 2-3 |
| Objectives | 4 | Provide an explicit statement of the objective(s) or question(s) the review addresses. | 3 |
| **METHODS** | | |  |
| Eligibility criteria | 5 | Specify the inclusion and exclusion criteria for the review and how studies were grouped for the syntheses. | 3-4 |
| Information sources | 6 | Specify all databases, registers, websites, organisations, reference lists and other sources searched or consulted to identify studies. Specify the date when each source was last searched or consulted. | 3-4 |
| Search strategy | 7 | Present the full search strategies for all databases, registers and websites, including any filters and limits used. | 3-4 |
| Selection process | 8 | Specify the methods used to decide whether a study met the inclusion criteria of the review, including how many reviewers screened each record and each report retrieved, whether they worked independently, and if applicable, details of automation tools used in the process. | 3-4 |
| Data collection process | 9 | Specify the methods used to collect data from reports, including how many reviewers collected data from each report, whether they worked independently, any processes for obtaining or confirming data from study investigators, and if applicable, details of automation tools used in the process. | 3-4 |
| Data items | 10a | List and define all outcomes for which data were sought. Specify whether all results that were compatible with each outcome domain in each study were sought (e.g. for all measures, time points, analyses), and if not, the methods used to decide which results to collect. | 3-4 |
|  | 10b | List and define all other variables for which data were sought (e.g. participant and intervention characteristics, funding sources). Describe any assumptions made about any missing or unclear information. |  |
| Study risk of bias assessment | 11 | Specify the methods used to assess risk of bias in the included studies, including details of the tool(s) used, how many reviewers assessed each study and whether they worked independently, and if applicable, details of automation tools used in the process. | 4-9 |
| Effect measures | 12 | Specify for each outcome the effect measure(s) (e.g. risk ratio, mean difference) used in the synthesis or presentation of results. |  |
| Synthesis methods | 13a | Describe the processes used to decide which studies were eligible for each synthesis (e.g. tabulating the study intervention characteristics and comparing against the planned groups for each synthesis (item #5)). |  |
|  | 13b | Describe any methods required to prepare the data for presentation or synthesis, such as handling of missing summary statistics, or data conversions. |  |
|  | 13c | Describe any methods used to tabulate or visually display results of individual studies and syntheses. |  |
|  | 13d | Describe any methods used to synthesize results and provide a rationale for the choice(s). If meta-analysis was performed, describe the model(s), method(s) to identify the presence and extent of statistical heterogeneity, and software package(s) used. |  |
|  | 13e | Describe any methods used to explore possible causes of heterogeneity among study results (e.g. subgroup analysis, meta-regression). |  |
|  | 13f | Describe any sensitivity analyses conducted to assess robustness of the synthesized results. |  |
| Reporting bias assessment | 14 | Describe any methods used to assess risk of bias due to missing results in a synthesis (arising from reporting biases). | 4-9 |
| Certainty assessment | 15 | Describe any methods used to assess certainty (or confidence) in the body of evidence for an outcome. | 4-9 |
| **RESULTS** | | |  |
| Study selection | 16a | Describe the results of the search and selection process, from the number of records identified in the search to the number of studies included in the review, ideally using a flow diagram. | 10 |
|  | 16b | Cite studies that might appear to meet the inclusion criteria, but which were excluded, and explain why they were excluded. | 10 |
| Study characteristics | 17 | Cite each included study and present its characteristics. | 11-12 |
| Risk of bias in studies | 18 | Present assessments of risk of bias for each included study. | 10-11 |
| Results of individual studies | 19 | For all outcomes, present, for each study: (a) summary statistics for each group (where appropriate) and (b) an effect estimate and its precision (e.g. confidence/credible interval), ideally using structured tables or plots. |  |
| Results of syntheses | 20a | For each synthesis, briefly summarise the characteristics and risk of bias among contributing studies. |  |
|  | 20b | Present results of all statistical syntheses conducted. If meta-analysis was done, present for each the summary estimate and its precision (e.g. confidence/credible interval) and measures of statistical heterogeneity. If comparing groups, describe the direction of the effect. |  |
|  | 20c | Present results of all investigations of possible causes of heterogeneity among study results. |  |
|  | 20d | Present results of all sensitivity analyses conducted to assess the robustness of the synthesized results. |  |
| Reporting biases | 21 | Present assessments of risk of bias due to missing results (arising from reporting biases) for each synthesis assessed. | 10-11 |
| Certainty of evidence | 22 | Present assessments of certainty (or confidence) in the body of evidence for each outcome assessed. | 11-12 |
| **DISCUSSION** | | |  |
| Discussion | 23a | Provide a general interpretation of the results in the context of other evidence. | 13-15 |
|  | 23b | Discuss any limitations of the evidence included in the review. | 15 |
|  | 23c | Discuss any limitations of the review processes used. | 13-15 |
|  | 23d | Discuss implications of the results for practice, policy, and future research. | 13-15 |
| **OTHER INFORMATION** | | |  |
| Registration and protocol | 24a | Provide registration information for the review, including register name and registration number, or state that the review was not registered. | 3 |
|  | 24b | Indicate where the review protocol can be accessed, or state that a protocol was not prepared. | 3 |
|  | 24c | Describe and explain any amendments to information provided at registration or in the protocol. |  |
| Support | 25 | Describe sources of financial or non-financial support for the review, and the role of the funders or sponsors in the review. | 16 |
| Competing interests | 26 | Declare any competing interests of review authors. | 16 |
| Availability of data, code and other materials | 27 | Report which of the following are publicly available and where they can be found: template data collection forms; data extracted from included studies; data used for all analyses; analytic code; any other materials used in the review. | 16 |

*From:*  Page MJ, McKenzie JE, Bossuyt PM, Boutron I, Hoffmann TC, Mulrow CD, et al. The PRISMA 2020 statement: an updated guideline for reporting systematic reviews. BMJ 2021;372:n71. doi: 10.1136/bmj.n71

**Full search terms**

Combining the keyword ‘COVID-19’ with the keywords ‘temperature’, ‘(meteorological variables)’, ‘(air pollutants)’, ‘(environmental variables)’, and ‘(socioeconomic variables)’, through the Boolean connector ‘AND’.

**Table S1.- List of studies excluded.**

**Only correlation**

Al-Rousan N, Al-Najjar H. The correlation between the spread of COVID-19 infections and weather variables in 30 Chinese provinces and the impact of Chinese government mitigation plans. *Eur Rev Med Pharmacol Sci.* 2020; 24(8):4565-4571. doi: <10.26355/eurrev_202004_21042>.

Bashir MF, Ma B, Bilal, Komal B, Bashir MA, Tan D, Bashir M. Correlation between climate indicators and COVID-19 pandemic in New York, USA. *Sci Total Environ.* 2020; 728:138835. doi: <10.1016/j.scitotenv.2020.138835>.

Beig G, Bano S, Sahu SK, Anand V, Korhale N, Rathood A, Yadav R, Mangaraj P, Murthy BS, Singh S, Latha R, Shinde R. COVID-19 and environmental – weather markers: Unfolding baseline levels and veracity of linkages in tropical India. *Environ Res.* 2020; 191:110121. doi: <10.1016/j.envres.2020.110121>.

Bennedeti F, Pachetti M, Marini B, Ippodrino R, Galloo RC, Ciccozzi M, Zella D. Inverse correlation between average monthly high temperatures and COVID-19-related death rates in different geographical areas. *J Transl Med.* 2020; 18(1):251. doi: <10.1186/s12967-020-02418-5>.

Bilal, Bashir MF, Benghoul M, Numan U, Shakoor A, Komal B, Bashir MA, Bashir M, Tan D. Environmental pollution and COVID-19 outbreak: insights from Germany. *Air Qual Atmos Health.* 2020; 1-10. doi: <10.1007/s11869-020-00893-9>.

Chatterjee A, Gerdes MW, Martínez SG. Statistical explorations and univariate timeseries analysis on COVID-19 datasets to understand the trend of disease spreading and death. *Sensors (Basel).* 2020; 20(11):3089. doi: <10.3390/s20113089>.

Mahahan UV, Larkins-Pettigrew M. Racial demographics and COVID-19 confirmed cases and deaths: a correlational analysis of 2886 US counties. *J Public Health (Oxf).* 2020; 42(3):445-447. doi: <10.1093/pubmed/fdaa070>.

Méndez-Arriaga F. The temperature and regional climate effects on communitarian COVID-19 contagion in Mexico throughout phase 1. *Sci Total Environ.* 2020; 735:139560. doi: <10.1016/j.scitotenv.2020.139560>.

Meo SA, Abukhalaf AA, Alomar A, Alsalame NM, Al-Khlaiwi T, Usmani AM. Effect of temperature and humidity on the dynamics of daily new cases and deaths due to COVID-19 outbreak in Gulf countries in Middle East Region. *Eur Rev Med Pharmacol Sci.* 2020; 24(13):7524-7533. doi: <10.26355/eurrev_202007_21927>.

Moges Menebo M. Temperatue and precipitation associate with COVID-19 new daily cases: A correlation study between weather and COVID-19 pandemic in Oslo, Norway. *Sci Total Environ.* 2020; 737:139659. doi: <10.1016/j.scitotenv.2020.139659>.

Nakada LYK, Urban RC. COVID-19 pandemic: environmental and social factors influencing the spread of SARS-CoV-2 in São Paulo, Brazil. *Environ Sci Pollut Res Int.* 2020; 1-7. doi: <10.1007/s11356-020-10930-w>.

Pani SK, Lin NH, RavindraBabu S. Association of COVID-19 pandemic with meteorological parameters over Singapore. *Sci Total Environ.* 2020; 740:140112. doi: <10.1016/j.scitotenv.2020.140112>.

Rendana M. Impact of the wind conditions on COVID-19 pandemic: A new insight for direction of the spread of the virus. *Urban Clim.* 2020; 34:100680. doi: <10.1016/j.uclim.2020.100680>.

Rouen A, Adda J, Roy O, Rogers E, Lévy P. COVID-19: relationship between atmospheric temperature and daily newcases growth rate. *Epidemiol Infect.* 2020; 148:e184. doi: <10.1017/S0950268820001831>.

Sahin M. Impact of weather on COVID-19 pandemic in Turkey. *Sci Total Environ.* 2020; 728:138810. doi: <10.1016/j.scitotenv.2020.138810>.

Sarmadi M, Marufi N, Moghaddam VK. Association of COVID-19 global distribution and environmental and demographic factors: An updated three-month study. *Environ Res.* 2020; 188:109748. doi: <10.1016/j.envres.2020.109748>.

Tello-Leal E, Macías-Hernández BA. Association of environmental and meteorological factors on the spread of COVID-19 in Victoria, Mexico, and air quality during the lockdown. *Environ Res.* 2021 (Epub 2020 Nov 11); 196:110442. doi: <10.1016/j.envres.2020.110442>.

Tosepu R, Gunawan J, Effendy DS, Ahmad OAI, Lestari H, Bahar H, Asfian P. Correlation between weather and COVID-19 pandemic in Jakarta, Indonesia. *Sci Total Environ.* 2020; 725:138436. doi: <10.1016/j.scitotenv.2020.138436>.

Tantrakarnapa K, Bhopdhornangkul B, Nakhaapakorn K. Influencing factors of COVID-19 spreading: a case study of Thailand. *Z Gesundh Wiss.* 2020; 1-7. doi: <10.1007/s10389-020-01329-5>.

Wang C, Li Z, Clay Mathews M, Praharaj S, Karna B, Solís P. The spatial association of social vulnerability with COVID-19 prevalence in the contiguous United States. *Int J Environ Health Res.* 2020; 23:1-8. doi: <10.1080/09603123.2020.1847258>.

Yaro CA, Udama Eneche PS, Anyebe DA. Risk analysis and hot spots detection of SARS-CoV-2 in Nigeria using demographic and environmental variables: an early assessment of transmission dynamics. *Int J Environ Health Res.* 2020; 1-12. doi: <10.1080/09603123.2020.1834080>.

Zoran MA, Savastru RS, Savastru DM, Tautan MN. Assessing the relationship between ground levels of ozone (O_3_) and nitrogen dioxide (NO_2_) with coronavirus (COVID-19) in Milan, Italy. *Sci Total Environ.* 2020; 740:140005. doi: <10.1016/j.scitotenv.2020.140005>.

**Descriptive studies (plots, maps)**

Baena-Díez JM, Barroso M, Cordeiro-Coelho SI, Díaz JL, Grau M. Impact of COVID-19 outbreak by income: hitting hardest the most deprived. *J Public Health (Oxf).* 2020; 42(2):698-703. doi: <10.1093/pubmed/fdaa136>.

Bontempi E. First data analysis about possible COVID-19 virus airborne diffusion due to air particulate matter (PM): The case of Lombardy (Italy). *Environ Res.* 2020; 186:109639. doi: <10.1016/j.envres.2020.109639>.

Bukkhari Q, Massaro JM, D’Agostino RB Sr, Khan S. Effects of weather on coronavirus pandemic. *Int J Environ Res Public Health.* 2020; 17(15):5399. doi: <10.3390/ijerph17155399>.

Caldas Dos Santos JP, San Pedro Siqueira A, Ferreira Praça HL, Gomes Albuquerque H. Vulnerability to severe forms of COVIID-19: an intra-municipal analysis in the city of Rio de Janeiro, Brazil. *Cad Saude Publica.* 2020; 36(5):e00075720. doi: <10.1590/0102-311x00075720>.

Chennakesavulu K, Ramanjaneya Reddy G. The effect of latitude and PM_2.5_ on spreading of SARS-CoV-2 in tropical and temperate zone countries. *Environ Pollut.* 2020; 266(Pt 3):115176. doi: <10.1016/j.envpol.2020.115176>.

Gunthe SS, Swain S, Patra SS, Amte A. On the global trends and spread of the COVID-19 outbreak: preliminary assessment of the potential relation between location-specific temperature and UV index. *Gesundh Wiss.* 2020; 1-10. doi: <10.1007/s10389-020-01279-y>.

Gupta S, Singh Raghuwanshi G, Chanda A. Effect of weather on COVID-19 spread in the US: A prediction model for India in 2020. *Sci Total Environ.* 2020; 728:138860. doi: <10.1016/j.scitotenv.2020.138860>.

Kumar S. Effect of meteorological parameters on spread of COVID-19 in India and air quality during lockdown. *Sci Total Environ.* 2020; 745:141021. doi: <10.1016/j.scitotenv.2020.141021>.

Martelletti L, Martelletti P. Air pollution and the novel COVID-10 disease: a putative disease risk factor. *SN Compr Clin Med.* 2020; 1-5. doi: <10.1007/s42399-020-00274-4>.

Ogen Y. Assessing nitrogen dioxide (NO(2)) levels as a contributing factor to coronavirus (COVID-19) fatality. *Sci Total Environ.* 2020; 726:138605. doi: <10.1016/j.scitotenv.2020.138605>.

Wadhera RK, Wadhera P, Gaba P, Figueroa JF, Joynt Maddox KE, Yeh RW, Shen C. Variation in COVID-19 hospitalizations and deaths across New York City boroughs. *JAMA.* 2020; 323(21):2192-2195. doi: <10.1001/jama.2020.7197>.

**Construction of an index**

Coccia M. An index to quantify environmental risk of exposure to future epidemics of the COVID-19 and similar viral agents: Theory and practice. *Environ Res.* 2020; 191:110155. doi: <10.1016/j.envres.2020.110155>.

Saha J, Chouhan P. Indoor air pollution (IAP) and pre-existing morbidities among under-5 children in India: are risk factors of coronavirus diseases (COVID-19)? *Environ Pollut.* 2020; 266(Pt 2):115520. doi: <10.1016/j.envpol.2020.115250>.

**Sensitivity, specificity, ROC**

Jahangiri M, Jahangiri M, Najafgholipour M. The sensitivity and specificity analyses of ambient temperature and population size on the transmission rate of the novel coronavirus (COVID-19) in different provinces of Iran. *Sci Total Environ.* 2020; 728:138872. doi: <10.1016/j.scitotenv.2020.138872>.

**Comparison of means, medians (without adjustment)**

Del Rio C, Camacho-Ortíz A. Will environmental changes in temperature affect the course of COVID-19? *Braz J Infect Dis.* 2020; 24(3):261-263. doi: <10.1016/j.bjid.2020.04.007>.

Livadiotis G. Statistical analysis of the impact of environmental temperature oon the exponential growth rate of cases infected by COVID-19. *PLoS One.* 2020; 15(5):e0233875. doi: <10.1371/journal.pone.0233875>.

**Fitting a distribution**

Huang Z, Huang J, Gu Q, Du P, Hongbin L, Dong Q. Optimal temperature zone for the dispersal of COVID-19. *Sci Total Environ.* 2020; 736:139487. doi: <10.1016/j.scitotenv.2020.139487>.

**Standardization**

Calderón-Larrañaga A, Vetrano DL, Rizzuto D, Bellander T, Fratiglioni L, Dekhtyar S. High excess mortality in areas with young and socially vulnerable populations during the COVID-19 outbreak in Stockholm Region, Sweden. *BMJ Glob Health.* 2020; 5(10):e003595. doi: <10.1136/bmjgh-2020-003595>.

**Principal component analysis**

Auler AC, Cássaro FAM, da Silva VO, Pires LF. Evidence that high temperatures and intermediate relative humidity might favor the spread of COVID-19 in tropical climate: A case study for the most affected Brazilian cities. *Sci Total Environ.* 2020; 729:139090. doi: <10.1016/j.scitotenv.2020.139090>.

**Odds ratio (without adjustment)**

Carta MG, Scano A, Lindert J, Bonanno S, Rinaldi L, Fais S, Orrù G. Association between the spread of COVID-19 and weather-climatic parameters. *Eur Rev Med Pharmacol Sci.* 2020; 24(15):8226-8231.doi: <10.26355/eurrev_202008_22512>.

**Table S2.- Studies included in the qualitative synthesis.**

| **First author** | **Study population** | **Study period** | **Outcome** | **Explanatory variables** | **Covariates** | **Statistical method** |
| --- | --- | --- | --- | --- | --- | --- |
| Aabed | Italy, Spain, China  World (188 countries) | April 13 | Cumulative cases Cumulative deaths | CO_2_ and proxies of air pollutants | Sea level  Population density | Fuzzy logic system |
| Adekunle | Africa (52 countries) | April 29 | Cumulative cases  Cumulative deaths | Temperature, relative humidity, wind speed |  | GAM with Gaussian lilnk |
| Adhikari | Queens, New York, USA | March 1-April 20 | New daily cases  Deaths | Temperature, relative humidity, precipitation, wind speed, cloud percentages  PM_2.5_, O_3_ |  | Negative binomial regression  Hurdle model for deaths |
| Ahmadi | Iran (31 provinces) | February 19-March 22 | Infection rate (number of infected people/days of infection) | Temperature, humidity, precipitation, wind speed, solar radiation | Population density, intra-provincial movement  infection days to  end of the study period | Linear regression |
| Azar | Northern California (21 counties, 10 of them in San Francisco Bay, Cohot, individual data) | January 1 – April 8 | Hospital admission of confirmed COVID-19 | Income of the ZIP code  Self-reported race and ethnicity, Primary insurance | Age, sex, comorbidities, smoking status | Logistic regression |
| Azuma | Japan (28 geographical areas) | March 13 – April 6 | New daily cases | Temperature, relative humidity, precipitation, wind speed, sunshine hours, vapor pressure  NO, NO_2_, photochemical oxidant, total suspended particulate matter, PM_2.5_ | Male inhabitants, Population density, percentage of inhabitants aged ≥65 years, total land surface area (km2), inhabitable area (km2), taxable income per person, tax debtor, life expectancy at birth, and health expenditure | Weighted random effects (errors normally distributed) |
| Behnood | USA (counties) | March | Infection rate (number of infected people/days of infection) | Temperature, precipitation | Population density | Machine learning method, the virus optimization algorithm (VOA) (errors normally distributed) |
| Briz-Redón | Spain (17 Autonomous Communities) | February 25 – March 28 | Cumulative cases | Temperature | Population density  Number of travellers  Number of companies | Spatio-temporal model (errors normally distributed) |
| Byass | China (grid cells) | January and February | New daily cases | Temperature, precipitation, solar radiation | Population density | Poisson regression |

| **First author** | **Study population** | **Study period** | **Outcome** | **Explanatory variables** | **Covariates** | **Statistical method** |
| --- | --- | --- | --- | --- | --- | --- |
| Carleton | World (3,235 geospatial units covering 173 countries) | January 1, April 10 | Daily growth rate of  confirmed cases | Temperature, specific humidity, precipitation, UV |  | Linear regression |
| Chadeau-Hyam | England (UK Biobank stuty – Cohort, individual data) | March 16-May 18 | Test (yes/no)  Positive/negative test | PM_10_, PM_2.5_, NO_x_  Socioeconomic variables | Lifestyle  Medical history and medications | Logistic regression |
| Chakrabarty | USA (all the states) | March 2 – April 30 | R0 | PM_2.5_ |  | GAM – Gaussian link |
| Chakraborty | USA (counties of the continental USA) | November 13 | Incidence | Ethnic groups, Socioeconomic deprivation, Persons (age 25+) with no high school diploma, Persons age 5+ speak English “less than well”, Persons below poverty, Civilians (age 16+) unemployed, Households with no vehicle available, Persons aged 65 and older, Civilian non-institutionalized with a disability, Civilian non-institutionalized with no health insurance, population density |  | Local Indicators of Spatial Association (LISA)  GEE – binomial link |
| Chaudhry | World | May 1 | Cumulative cases,  Cumulative recovered cases  Cumulative critical cases  Overall mortality  all expressed per million population | GDP per capita based on purchasing power parity, median population age,  gender distribution of population (%), population density, unemployment rate, Corruption  Perceptions Index, Gini index, Prevalence of obesity | Public health policies for each country | Negative binomial model |
| Chien | USA (top 50 most prevalent counties) | March 16-April 22 | New daily cases | Temperature, humidity, precipitation | Median age, male percentage,  white percentage, black percentage, hispanic  percentage, and poverty percentage | GAM – Poisson link |
| Coccia a | Italy (55 cities) | February and March | Cumulative cases | Temperature, precipitation wind speed, moisture, fog  PM_10_, O_3_  Population density |  | Linear regression |

| **First author** | **Study population** | **Study period** | **Outcome** | **Explanatory variables** | **Covariates** | **Statistical method** |
| --- | --- | --- | --- | --- | --- | --- |
| Coccia b | Italy (55 citiies) | April 7 | Cumulative cases  Deaths | Average wind speed  PM_10_, O_3_ | Density of population; elderly people index; people aged >65 years old  Mortality rate of trachea, bronchi and lung cancer; Rate of mortality for diseases of respiratory system; Rate of mortality for diseases of cardiovascular system | Linear regression |
| Coker | Italy (municipalities of 8 regions of Nothern Italy) | January 1-April 30 | Difference between the number of deaths in the period January 1—April 30, 2020, and the average number of deaths in the same period of  the previous 5 years | Temperature  PM_2.5_  Percentage of males, percentage of population 65 years old and older, Population density, per-capita income, shares of municipality area occupied by industrial sites, average size of manufacturing  Firms, share of non-EU citizen, percentage of university students on the total population, distance from the closest airport, number of  hospital beds |  | Negative binomial regression |
| Das a | Chennai megacity (155 electoral wards), India | May 15-May 21 | Cumulative cases | Index of Multiple Deprivation |  | Poisson regression  Negative binomial regression |
| Das b | Kolkata megacity, India | NA | Cumulative cases | Urban patch density, land surface temperature, normalized differential vegetation index, normalized differential water index, normalized differential moisture index, index of multiple deprivation, population density, household density | Housing condition, asset possession, wash services, household amenities and services, gender disparity | Poisson regression  Negative binomial regression Hurdle regression  Zero-inflated Poisson  Zero-inflated negative binomial |

| **First author** | **Study population** | **Study period** | **Outcome** | **Explanatory variables** | **Covariates** | **Statistical method** |
| --- | --- | --- | --- | --- | --- | --- |
| Demongeot | France (13 regions)  World (21 countries) | February 1 – March 14 | New daily cases | Temperature |  | Exponential regression model  ARIMA models  (in both, errors normally distributed) |
| DiMaggio | New York (USA) | April 22 | Total number of positive test  Total number of tests performed | Total population, proportion of persons older than 65 years, number of persons self-identifying as Black/African American, Asian or Hispanic, number of persons older than 5 years, speaking a language other than English, population density, housing density, school density, proportion of persons receiving public assistance |  | Spatial model (Besag, York, Mollié) |
| Dogan | New Jersey (21 counties), USA | March 1 – July 7 | New daily cases | Temperature,humidity  PM_2.5_, Air quality index |  | Linear regression |
| Drefahl | Sweden (individual data) | March 13 - May 7 | Deaths | Age, sex, civil status, education, individual net income, country of birth, county of residence |  | Survival analysis |
| Falcão Sobral | World (does not indicate which countries) | January 12 – March 30 | New daily cases  Deaths | Temperature, precipitation | Population density, time exposure to the disease | Panel data (errors normally distributed) |
| Fattorini | Italy (71 provinces) | NA – April 27 | Cumulative cases | NO_2_, PM_10_, PM_2,5_, O_3_ |  | Linear regression |
| Fazzini | Lombardy, Italy | March 1, April 20 | Ratio between the first positive swabs and the total number of swabs collected per day | Temperature, relative humidity, precipitation, wind speed, evaporation |  | Linear regression |
| Fiasca | Italy (71 provinces) | March-October | Incidence rate | PM_2.5_, NO_2_ | Old-age index  Population density | Linear regression |

| **First author** | **Study population** | **Study period** | **Outcome** | **Explanatory variables** | **Covariates** | **Statistical method** |
| --- | --- | --- | --- | --- | --- | --- |
| Filippini | Italy (Provinces of Lombardia, Veneto, Emilia-Romagna) | March 8, 22 and April 5 | Prevalence | NO_2_ | Temperature, dewpoint temperature, relative humidity  Population density, Ratio between resident population aged  ≥65 years and those aged ≤14 years  Mobility indicators  Single member families or dwellings occupied by only one resident. | Restricted cubic spline regression analysis  (errors normally distributed) |
| Fu | Europe (42 provincial regions of France, Germany, Italy, Spain) | Febr. 1-November 1 | Time it takes for the total number of COVID-19 cases to double | Average/minimum/maximum temperature, dew-point temperature, average wind velocity, absolute humidity | Population density  PM_2.5_  GRI – government response index (measures of policies) | Poisson regression |
| Guasp | World (359 countries and regions of China, Italy, USA, Spain, Canada and Australia) | After determining  the date in which the number of confirmed cases was ≥ 10 for  each of the studied regions, we defined the beginning of the  study period at day −7 to account for the incubation period of  around 1 week and prolonged it until day +30 or the day of the  analysis (23 March 2020) if shorter | Cumulative incidence (per 100,000 inhabs) | Temperature, relative  humidity, precipitation, dew point, pressure, wind speed, wind direction, cloud coverage,  snowfall snow depth, diffuse and global horizontal solar irradiance, maximum ultraviolet (UV) index | Population density  Continent of each country and country of each region used as controls | Linear regression |
| Guo C | World (235 cities from 10 countries and 180 countries) | January 23 – April 13 | New daily cases | Temperature, relative humidity, wind speed | Population size, population density, median age, Global Health Security Index, public health interventions | Negative binomial regression |

| **First author** | **Study population** | **Study period** | **Outcome** | **Explanatory variables** | **Covariates** | **Statistical method** |
| --- | --- | --- | --- | --- | --- | --- |
| Guo XJ | China (4 cities) | January 24 – Febr. 13 | Cumulative cases | Temperature, relative humidity, absolute humidity |  | Linear regression |
| Gupta | 9 cities of India, China, Pakistan, Indonesia | NA – July 2 | Cumulative deaths divided by the cumulative cases | PM_2.5_, PM10 |  | Linear regression |
| Han | Beijing, China | June 11 – July 5 | New daily cases | Distance to traffic facilities  Distance to parks  Distance to Xinfadi market  Distance to business sites  Distance to educational facilities  Distance to shopping sites  Distance to restaurants  Distance to hospital |  | Geographically weighted regression  (errors normally distributed) |
| He | Asia (9 cities) | January 20-March 18 | New daily cases | Temperature, relative humidity |  | GAM - Poisson link |
| Hoang a | Korea (7 metropolitan cities and 9 provinces) | February 24 – May 5 | New daily cases | Temperature, relative humidity, wind speed, air pressure  PM_10_, PM_2.5_, NO_2_, O_3_, CO, SO_2_ |  | GAM - Gaussian link |
| Hoang b | South Korea (two clusters) | Febr. 24 – Sept. 12 | New daily cases | PM_2.5_, PM_10_, O_3,_ NO_2_, CO, SO_2_ | Temperature, humidity, wind speed, air pressure | GAM – Gaussian link |
| Hutter | Vienna, Austria (population of 23 districts, individual data) | Date of the first diagnosed case in the district – Diagnosis, death, or 21 April 2020 (whichever came first) | New daily cases  Deaths | PM_10_, NO_2_ | Percentage of population aged 65 and more; Percentage of foreigners; Percentage of persons with a university degree, Unemployment rate, Population density | Cox regression |
| Iqbal MM | World (210 countries) | 1^st^ reported case - June 5 | Cumulative cases  Deaths | Temperature, Daylight hours |  | Linear regression |
| Iqbal N | Wuhan, China | January 21 – March 31 | New daily cases | Temperature | Chinese exhange rate | Wavelet methods (errors normally distributed) |

| **First author** | **Study population** | **Study period** | **Outcome** | **Explanatory variables** | **Covariates** | **Statistical method** |
| --- | --- | --- | --- | --- | --- | --- |
| Isaia | Italy (20 regions) | February 25 – May 31 | Cumulative cases  Deaths  Swabs  (all per 100,000 inhabitants) | Ultraviolet exposure, temperature, relative humidity | PM_10_  Mortality rates of comorbidities (isheamic heart, circulatory system disease, cerebrovascular disease, DM2)  Average mortality rate, mean age, nursing home residents per 100,000 inhab. | Linear regression |
| Islam ART | Bangladesh (districts) | March-April | New daily cases  Deaths | Temperature, relative humidity, absolute humidity |  | Compound Poisson-Gamma regression  Montecarlo methods  Random forests |
| Islam N | World (regions/countries that reported ≥100 COVID-19 cases) | April 29 | Cumulative cases | Temperature, relative humidity, UV index, wind speed, cloud cover, precipitation, sea-level air-pressure, daytime length | GDP per capita, Global Health Security Index | Multilevel mixed-effects negative binomial model |
| Jamshidi | World  USA counties | January 1 – August 15 | New weekly cases | Equivalent temperature (combines temperature and relative humidity)  Urban density, mobility (number of trips, 1-100 miles per day; percentage change 2020-2019 in the number of trips, homestay); mask usage |  | Linear regression |
| Jiang | China (3 cities) | January 25 – Febr. 29 | New daily cases | Temperature, relativity humidity, wind level  PM_2.5_, PM_10_, SO_2_, CO, NO_2_, O_3_ |  | Poisson regression |

| **First author** | **Study population** | **Study period** | **Outcome** | **Explanatory variables** | **Covariates** | **Statistical method** |
| --- | --- | --- | --- | --- | --- | --- |
| Jüni | World (144 areas, excluding China, South Korea, Italy, Iran) | March 20 – March 27 | Cumulative cases on March 27/Cumulative cases on March 20 | Temperature, absolute humidity, Latitude | School closures, restrictions of mass  gatherings, and measures of social distancing  Altitude, gross domestic product (GDP) per capita, health expenditure  as percent of GDP, life expectancy, percentage of inhabitants  aged 65 years or older, the Infectious Disease Vulnerability  Index, urban population density, number of flight passengers  per capita and closest distance to a country with already established  epidemic (city of Wuhan, South Korea, Iran, Italy) | Weighted random effects (errors normally distributed) |
| Kaiser | World (21 countries) | April 11,16 and May 12 | Cumulative cases  Cumulative deaths | Population, urban population, median population age, population density  Inflation-adjusted health expenditure  Annual gross domestic product | H1N1 cases | GLM – Gaussian link |
| Khan | China (prefectures on mainland China) | NA- April 3 | Rates of new daily cases, cured and death  R0 | Temperature |  | Stochastic transmission dynamic model  (errors normally distributed) |
| Kodera | Japan (14 prefectures) | May 25 | Cumulative cases  Cumulative deaths | Temperature, absolute humidity, wind velocity, daylight  Population density, percentage of elderly population |  | Linear regression |
| **First author** | **Study population** | **Study period** | **Outcome** | **Explanatory variables** | **Covariates** | **Statistical method** |
| Kubota | World (1020 countries/regions) | December 2019- June 30 | New daily cases per million inhabitants | Temperature, precipitation  Population size, population  density  GDP, GDP per person | COVID-19 susceptibility (BCG vaccination factors, malaria  incidence, and percentage of the population aged over 65 years), and human mobility (relative  amounts of international visitors  To control for country/region-specific observation biases, it is included the length of time  (measured in days) since the first confirmed COVID-19 case in each country/region and the  number of COVID-19 tests conducted (as a measure of sampling effort) as covariates | Linear regression |
| Lamb | New York city, USA (177 ZIP codes) | April 1 | Proportion of COVID-19 tests found positive | Proportion of the 18- to 64-year-old population that is uninsured  Median household income  Proportion of population that self-identified their race as white  Proportion of population living in households with more than  three inhabitants  Proportion of population using public transportation to commute  to work that includes bus travel  Proportion of population that is elderly (65+ years of age) |  | GEE – Gaussian link |
| Lau | China (all provinces of China) | January 1 – February 5 | Cumulative cases | number of domestic passengers (air traffic), number of international routes,  number of international passengers, number of  foreign cities with international air travel with China |  | Linear regression |
| **First author** | **Study population** | **Study period** | **Outcome** | **Explanatory variables** | **Covariates** | **Statistical method** |
| Lhada | Delhi, India | April 1- May 31 | New daily cases | Temperature, relative humidity | Number of COVID-19 tests performed | Linear regression |
| Li AY | USA (all counties) | April 14 | Cumulative cases, Cumulative deaths | Temperature  Race demographics by county, County GDP, Population Demographics, Health, and Social Determinants  of Health Statistics  Change in average distance travelled was used as an  indicator of social distancing  Data from mobile phones  was used to track average changes in distance travelled  within counties after the first reported COVID-19 case | Diabetes, Hypertension,  Coronary Artery Disease, Obesity, Poverty, Pollution, Respiratory mortality, Liver mortality | Linear regression  Logistic regression (to compare counties in the highest  quartile for cases/100,000 to those in the lowest) |
| Li H | China (2 cities) | Febr. 26-Februaruy29 | New daily cases | Temperature, sunrise duration  PM_2.5_, PM_10_, NO_2_, CO, Air Quality Index |  | Linear regression |
| Li X | Wuhan, China | January 23 – Febr. 13 | Cumulative cases | urban growth, general hospital, commercial facilities, subway station, land-use mixture, aging ratio, road density |  | Linear regression  Geographically weighted regression model (GWR)  (errors normally distributed) |
| Liang | USA (3122 counties) | January 22 – July 17 | Case-fatality rates  Death rates | NO_2_, PM_2.5_, O_3_ | Temperature, relative  humidity  County-level health care capacity, population mobility,  population density, socioeconomic status index (SES),  race and ethnicity, behavior risk factors  State-level COVID-19 test positive rate | Zero-inflated negative binomial |

| **First author** | **Study population** | **Study period** | **Outcome** | **Explanatory variables** | **Covariates** | **Statistical method** |
| --- | --- | --- | --- | --- | --- | --- |
| Lin | World (69 countries) | March 17 | Cumulative cases | Temperature, relative humidity | Population of each country  Number of hospital beds, number of nurses, and number of physicians per 1000 | First stage: using individual data from China (11 mainland provinces), Hong Kong, Singapore 🡪 extended SEIR  Second stage: using the transmission rates of the provinces in mainland China and the doubling time of confirmed cases in 69 countries outside China, together with the daily temperature and relative humidity 🡪 Multiple regression |
| Liu | China (30 cities) | January 20 – March 20 | New daily cases | Temperature, absolute humidity | Migration scale index | First stage. GLM negative binomial  Second stage. Meta-analysis |
| López-Feldman | Mexico city (individual data) | NA | Death | PM_2.5_ | Municipal level: population  size, population density, hospital beds per capita, poverty  measures, percentage of jobs that can be done at home.  Individual level:  sex, obesity, smoking status, diabetes, hypertension, age, age squared, day in which symptoms started | Probit |
| Luo | USA (3108 counties of the continental USA) | January 22-June 26 | Death rate | Atmosphere, climate, land cover  socioeconomic and demographic factors | Disaster, health status, commuting to work | Geographically weighted random forest (errors normally distributed) |
| Ma | Wuhan, China | January 20 – Febr. 29 | Deaths | Temperature, relative humidity, absolute humidity  PM_2.5_, PM_10_, SO_2_, CO, NO_2_, O_3_ |  | GAM – quasi Poisson link |
| Madhav | Lousiana, USA (1148 census tracts) | July 31 | Cumulative cases | ADI (deprivation index)  17 socioeconomic variables  Indicator rural/urban |  | Poisson regression |
| **First author** | **Study population** | **Study period** | **Outcome** | **Explanatory variables** | **Covariates** | **Statistical method** |
| Malki | World (Italy -21 states-; United States -3144 counties and 5 territories-; country-level aggregates for the rest of the  world) | December 12-April 22 | Cumulative cases Cumulative deaths  Cumulative recovered Active cases | Temperature, humidity,  precipitation, snowfall, moon illumination, sunlight hours,  ultraviolet index, cloud cover, wind speed and direction, pressure | Population density | Machine learning models  (errors normally distributed) |
| Mandal | World (>200 countires) | March 25 – April 18 | Cumulative cases | Temperature |  | Log-linear regression  (errors normally distributed) |
| Marciel de Souza | Brazil (national level) and states (São Paulo, Rio de Janeiro, Ceará and Amazonas)  Ecological and individual  The analysis  was replicated on equivalent time series from Italy, Spain, France and the United  Kingdom  Ecological | February 25-May 31 | Reproductive number  COVID-19 case and severe acute respiratory infection cases with  unknown aetiology | Household per-capita income |  | Logistic regression |
| Martorell-Marugan | Spain (17 Autonomous Communities, 2 Autonomous Cities, 52 provinces) | January 1 -… | New daily cases  Daily deaths  Cumulative cases  Positive PCR | Temperature, rainfall,  wind speed, solar radiation  NO_2_, CO, PM_2.5_, PM_10,_ SO_2,_ O_3_ |  | Linear regression |
| Medeiros-Figuereido | Spain (17 Autonomous Communities) | March 15 – April 22 | Cumulative cases  Cumulative deaths | Temperature | Population density  Proportion of people over 65, ratio of residential places per 100 people over 65 years  Doctors per 1,000 inhabitants, beds  hospital and ICU beds per 100,000 people | Linear regression |
| Meo | World (top ten hottest and top ten coldest countries) | December 29, 2019 – May 12 | Daily cases  Daily deaths  Cumulative cases  Cumulative deaths |  |  | Linear regression |
| **First author** | **Study population** | **Study period** | **Outcome** | **Explanatory variables** | **Covariates** | **Statistical method** |
| Meraj | India (3 regions) | March 9 – May 27 | Cumulative cases | Temperature |  | Linear regression |
| Meyer | World | December 29, 2019- March 18 | New daily cases | Temperature, relative humidity | Median age of the national population, age-structure, population density  Global Health Security Index | GLMM  Negative binomial model |
| Muñoz-Cacho | Spain (17 Autonomous Communities) | March 1 - March 31 | Cumulative  incidence during the previous 14 days  Total cases  Nwly diagnosed cases Hospital admissions  ICU admissions  Cumulative deaths | Ultraviolet radiation, temperature, relative humidity |  | Linear regression |
| Notari | World (88 countries) | March 26, April 1, 14 | Cumulative cases | Temperature |  | Linear regression |
| Ozyigit | World (15 European Countries) | 60 days since each country’s 100th reported case | Rate of growth of the daily case numbers | Temperature | Average years of schooling, length of time to government response | Panel data  (errors normally distributed) |
| Paez | Spain (52 provinces) | March 13-April 11 | New daily cases | Temperature, relative humidity, sunshine | GDP per capita, Percentage of people aged 65 and older, Population density | SUR-SLM  SUR: Seemingly Unrelated Regression equations model  (errors normally distributed) |
| Pan | World (202 locations in 8 countries) | NA | R0 | Temperature, relative humidity, wind speed ultraviolet radiation |  | Linear regression  SEIR models  (errors normally distributed) |
| Pequeno | Brazil (27 state capital cities) | February 26-March 26 | Cumulative cases | Temperature, relative humidity, precipitation, solar radiation | Number of arriving  flights in the city’s metropolitan area in 2020  Population density; proportion of elderly  people (≥60 years old); citizen mean income | GLMM Poisson link |

| **First author** | **Study population** | **Study period** | **Outcome** | **Explanatory variables** | **Covariates** | **Statistical method** |
| --- | --- | --- | --- | --- | --- | --- |
| Perone | Italy (20 regions and 107 provinces) | April 3 and 4, 2020 | Average case fatality rate (average  confirmed deaths by the average confirmed cases) | Temperature, humidity  PM_10_, PM_2.5_, NO_2_, O_3_  Health system metrics  Demographic variables, Three ad hoc indexes that represent the health system saturation |  | Linear regression |
| Pirouz | Italia (Lombardy, Veneto, Emilia-Romagna) | Febr. 14 – March 14 | New daily cases | Temperature, relative humidity, wind speed | Total number of the confirmed cases in 14 days | Linear regression |
| Plümper | Germany (401 local districts) | April 13-May 19 | Cumulative cases  New infected  Cumulative deaths  New deaths | Taxable income, unemployment rate, percentage of workforce university-educated, urban district dummy, very remote district dummy, population share catholic |  | Linear regression |
| Poirier | China  World (Iran, Italy, Singapore, Japan, South Korea) | January 22 – Febr. 26 | Reproductive number | Temperature, absolute humidity | Mobility (percentage of people traveling from Wuhan and going to the different Chinese provinces) | Linear regression |
| Pozzer | World | NA | Mortality | Particulate air pollution |  | Exposure–  response function of the WHO  Estimation of the attributable fraction |
| Pramanik | Rusia | NA | Cumulative cases | Temperature, relative humidity, sunshine, wind speed |  | Random forest  (errors normally distributed) |
| Pramanik | World (228 cities) | May 25 | Cumulative cases | Temperature, relative humidity, sunshine, wind speed |  | Bosted regression trees  (errors normally distributed) |
| Prata | Brazil (all state capitals) | February 27 – April 1 | Cumulative cases | Temperature | Population density, population, counting days since the first outbreak | GAM – Poisson link |

| **First author** | **Study population** | **Study period** | **Outcome** | **Explanatory variables** | **Covariates** | **Statistical method** |
| --- | --- | --- | --- | --- | --- | --- |
| Price-Haywood | Ochsner Health facility, New Orleans, Lousiana, USA(Cohort, individual data) | March 1 – April 1 | Patients who tested positive for SARS-CoV-2 on | Demographic characteristics; chronic conditions, body-mass index, selected outpatient medications, symptoms and diagnosis codes linked to primary care, emergency department or urgent care encounter during which Covid-19 testing occurred,  vital signs, medications, and laboratory or procedure codes and diagnoses linked to inpatient encounters | Percentage of low-income residents in ZIP codes that exceeds the Lousiana benchmarks (39.5%) | Logistic regression |
| Qi | China (all provinces of China) | December 1, 2019 – February 11 | New daily cases | Temperature, relative humidity | Baidu Index with massive Internet behavior data recorded | GAM – negative binomial link |
| Rafael | Rio de Janeiro, Brazil | April 7-April 13 | Cumulative incidence rate | Per capita income  Percentage of women in the population, the proportion of elderly individuals above the age of 60, and the percentage of afro-descendent and mixed-race individuals |  | Quantile regression  (errors normally distributed) |
| Rahman | World (149 countries) | January 1 - May 10 | Cumulative cases  Cumulative deaths  Cumulative tests performed | Temperature  Population density, GDP growth  rate, GDP per capita, population growth rate, life expectancy, percentage of population  over 60 years, health expenditure, physicians per thousand people and environmental  variables including number of threatened species, forested area, CO_2_ emission, protected area percentage of the forested area |  | Linear mixed effect models  (errors normally distributed) |
| Rashed | Japan (16 prefectures) | March 15-May 25 | New daily cases | Temperature, absolute humidity | Population density | Linear regression |

| **First author** | **Study population** | **Study period** | **Outcome** | **Explanatory variables** | **Covariates** | **Statistical method** |
| --- | --- | --- | --- | --- | --- | --- |
| Rehman | Pakistan (7 regions) | March 10 – July 10 | New daily cases,  Deaths  Recovery and daily testing | Temperature, humidity wind and sun status | lock down status and maximum daily testing status | GLMM - negative binomial link |
| Richmond | Georgia, USA (159 counties) | April 1, May 1, June 1, July 1, August 1 | Rate of cumulative cases (per 100,000 inhabs.) | Percent with long commute who drive alone, Percent non-Hispanic White residents, Percent of children qualifying for free lunch, Percent who report poor or fair health, Percent not proficient in English, Segregation index: Black/White, Percent of uninsured adults, Percent female, Percent with annual influenza vaccine, Teen birth rate, Percent under 18 years of age, child mortality rate |  | Linear regression  Penalized regression  (errors normally distributed) |
| Rodríguez-Villamizar | Colombia (772 municipalities) | NA – July 17 | Death rates | PM_2.5_ | Percentage of population 65 years or older, percentage of the urban population, population density, poverty index, hospital beds capacity,  number of COVID-19 tests at the department level, and prevalence  of hypertension, diabetes, and chronic renal failure. | Hurdle models (logistic regression-negative binomial) |
| Rozenfeld | Providence Health System, Providence, USA (Cohort, individual data) | February 28 – April 27 | COVID-19 infection (positive laboratory test) | Physical proximity to other people, transportation insecurity, relationship status, employment, housing insecurity, and age-stratified communal living.  Age, BMI, number of medications, and neighborhood financial insecurity, Comorbidities,  Substance, tobacco, and alcohol consumptions |  | Logistic regression |

| **First author** | **Study population** | **Study period** | **Outcome** | **Explanatory variables** | **Covariates** | **Statistical method** |
| --- | --- | --- | --- | --- | --- | --- |
| Rubin | United States (211 counties) | February 25 – April 23 | Reproductive number | Wet-bulb temperature | Social distance (daily cellular telephone movement), population density  Demographic factors (eg, age distribution, insurance status, and  socioeconomic status) Health-related factors associated with COVID-19 severity (eg, proportion of individuals with hypertension, obesity, or diabetes and proportion of individuals who smoke) | Linear mixed-effects model  (errors normally distributed) |
| Runkle | United States (8 cities) | NA | New daily cases | Temperature, specific humidity, solar radiation |  | Case crossover - Poisson regression |
| Saez | Catalonia, Spain (health regions and counties) | February 25 – May 16 | Daily positive cases Daily deaths | PM_10_, NO_2_ | Average income per person; unemployment rate; Percentage of population aged 65 and over; Percentage of foreigners in 2019 from countries with medium and low human development index according to the United Nations Development; Poor housing; Percentage of single person households; Population density | GLMM – Poisson link |
| Sajadi | World (50 cities) | NA – March 10 | Deaths | Temperature, relative humidity, specific humidity, absolute humidity |  | Linear regression |

| **First author** | **Study population** | **Study period** | **Outcome** | **Explanatory variables** | **Covariates** | **Statistical method** |
| --- | --- | --- | --- | --- | --- | --- |
| Sánchez-Lorenzo | Europe (15 capital cities) | March 26 | Cumulative cases | Atmospheric data NCEP/NCAR reanalysis (for Europe)  For Spain: Temperature, air pressure, wind speed, specific humidity, relative humidity, total precipitation, and days of more than 1 mm of precipitation |  | Linear regression |
| Sannigrahi | Europe | December 31-April 29 | Cumulative cases  Cumulative deaths  All per 100,000 habs. | 28 socio-demographic variables |  | Linear regression  Spatial regression models, Spatial Lag Model  (errors normally distributed) |
| Sarkodie | World (20 countries) | January 22 – April 27 | New cases  Daily deaths  Daily recovered | Temperature, relative humidity, wind speed, Dew/Frost point, surface pressure |  | Panel data |
| Scarpone | Germany (counties) | January 28-March 31 | Age-adjusted incidence rates | Church density, voter turnout, Proportion of foreign guests  in overnight stays, Driving distance to IC/EC/ICE, Employment rate, Long−term unemployment rate, Unemployment rate (Age <25), Regional population potential |  | GAM – Gaussian link  Geospatial analysis  Bayesian Machine Learning  Bayesian Additive Regression Trees |
| Sehra | USA (all the states) | January 22 – April 3 | New daily cases | Temperature, precipitation, UV index | Population density, population, African american percentage, older adult percentage, obesity rates, state GDP, percentage of child, median family income, rates of uninsured, graduation rates | Negative binomial – GEE |
| Shahzad F | China (10 provinces) | January 22 – March 31 | Cumulative cases | Temperature |  | Quantile on quantile regression  (errors normally distributed) |
| Shahzad K | Spain (4 regions) | February 29 – July 17 | Daily cases | Temperature  PM_2.5_ |  | Panel regression,  Quantile regression  (errors normally distributed) |
| Shao | World (47 countries) | February 22 – June 22 | Effective reproductive number | Temperature, air pressure, wind speed | Mobility (driving, walking and transit) | Panel data  (errors normally distributed) |
| Shi | China (31 provinces) | January 20 – Febr. 29 | New daily cases | Temperature |  | GAM – quasi Poisson link  Meta-analysis |
| **First author** | **Study population** | **Study period** | **Outcome** | **Explanatory variables** | **Covariates** | **Statistical method** |
| Stieb | Canada (111 health regions) | NA-May 13 | Number of cumulative cases | Temperature  PM_2.5_ | Population density, Percent age 65+, Percent < Low Income Cut-off, Percent black, percent asthma, percent COPD, percent hypertension, percent diabetes, percent physically active, percent overweight, percent obese, percent smokers, days since first case, days since peak incidence, Normalized Difference Vegetation Index (NDVI) | Negative binomial models |
| Su | World (227 regions) | Dec. 1 2019 – April 14 | Time-dependent transmission rate | Humidity, temperature, wind speed, and visibility data  Location, population, people aged over 65, area, life expectation , the number of hospital bed, GDP | Dates when strict control policies were implemented, typically border closure, strict travel restrictions/ban, and/or a state of emergency was declared | Linear regression |
| Sun | England (local authorities districts) | March, April, May | Death rates | Temperature, relative humidity  Percentage of females, percentage of asians, percentage of blacks, Percent of households in poverty, Unemployment rate, Density of population, Density of hospital | PM2.5 (only control) | Spatial regression models  (errors normally distributed) |
| Tagaki a | United States (33 largest cities) | NA- May 14 | Cumulative cases | Temperature, relative humidity, precipitation, wind speed, sky cover |  | Random effects meta-regression  (errors normally distributed) |
| Tagaki b | Japan (prefectures) | January-April | Cumulative cases | Temperature, wind speed, sea level air pressure, relative  humidity, possible sunshine, daily maximum UV index; total of sunshine duration, precipitation |  | Meta-regression  (errors normally distributed) |
| To | Canada (4 provinces) | January-May | Reproductive number  Cumulative incidence | Temperature, wind speed, precipitation |  | Linear regressioon |
| **First author** | **Study population** | **Study period** | **Outcome** | **Explanatory variables** | **Covariates** | **Statistical method** |
| Tobías | Barcelona’s Health Region, Spain | March 2 – April 25 | New daily cases | Temperature | Weekends, lockdown period | GLM – quasi Poisson link |
| Tzampoglou | World | March-May | Cumulative incidence Death rates | Temperature, relative humidity, precipitation, cloud cover | Population density, Percentage of population over 65  Median age and the government response measures, Human Development Index (HDI) | Linear regression |
| Ujiie | Japan (47 prefectures) | January 15 – March 16 | Cumulative cases per 1,000,000 population | Temperature | Number of  inbound visitors from China in January 2020, old-age dependency ratio (the ratio of the number of people aged 65 and over, compared to the number of people 15-64 years old) | Poisson regression |
| Wang Q | China (28 Chinese provincial regions) | January 20 – Febr. 29 | Instantaneous reproductive number | Temperature, relative humidity, air pressure, wind speed |  | GAM – Gaussian link |
| Wang Y | China (Hubei and Hunan provinces) | NA | Deaths | NO_2_ |  | GAM - Gaussian link |
| Ward a | New South Wales, Australia | January to March | New daily cases | Temperature, relative humidity, precipitation |  | GAM – Negative binomial link |
| Ward b | New South Wales, Australia | January to May | New daily cases | Temperature, relative humidity, precipitation, wind speed |  | GAM – quasi Poisson link |
| Wu X | USA (3,087 counties) | Up to and including June 18 | Deaths | PM_2.5_  Socioeconomic and demographic variables  Meteorological variables (summer temperature and relative humidity, winter temperature and relative humidity) | 20 county-level covariates in the main model  two county-level health risk factors  county’s point on the epidemic curve at the time of analysis | Negative binomial mixed models  More than 80 sensitivity analyses, including ZIP, etc. |
| **First author** | **Study population** | **Study period** | **Outcome** | **Explanatory variables** | **Covariates** | **Statistical method** |
| Wu Y | World (166 countries) | January 1 – March 27 | New daily cases | Temperature, relative humidity, wind speed | Median age of population, Global Health Security Index, country, date of the week | GAM – Gaussian link |
| Xie J | China (122 cities) | January 23 – Febr. 29 | New daily cases | Temperature, relative humidity, air pressure, wind speed | Day, city | GAM – Poisson link |
| Xie Z | China (mainland) | January 24 – Febr. 20 | Cumulative cases | Temperature  Population density, Proportion of incoming population in Wuhan, Distance from Wuhan, Strength of economic connection with Wuhan, Number of hospital beds per 1000 persons |  | Spatial differentiation (Geodetector method)  (errors normally distributed) |
| Xu | China (33 locations) | January 29 – Febr. 15 | Cumulative cases | Air quality  Temperature, relative humidity, wind speed, pressure |  | Poisson regression |
| Yao a | China (224 cities, 17 of Hubei) | February 10 – March 9 | Cumulative cases  Basic reproduction number | Temperature, relative humidity, UV radiation |  | Linear regression |
| Yao b | China (63 cities) | NA | R0 | PM10, PM2.5, NO2, SO2, CO, O3 | Temperature, relative humidity  Population density of population, GDP per capita, hospital beds | Linear regression |
| You | Wuhan (13 districts), China | NA | Cumulative cases | Population density, Construction land area proportion, Average building scale, GDP per unit of land area, Value-added of tertiary industry per unit of land area, Total retail sales of consumer  goods per unit of land area, Public green space density, Hospital density, Aged population density | Correlation (Pearson) | Spatial regression  (errors normally distributed) |

| **First author** | **Study population** | **Study period** | **Outcome** | **Explanatory variables** | **Covariates** | **Statistical method** |
| --- | --- | --- | --- | --- | --- | --- |
| Zakeri | London (Case-control, individual ddata) | March 1- June 2 | City adult residents admitted to hospital with confirmed COVID-19 were compared with matched controls randomly sampled from a primary healthcare database  comprising 344,083 people residing in the same region.  Cohort study  we studied 1827 adults consecutively  admitted with COVID-19 | Deprivation index  Ethnic groups | Age, sex, comorbidities | Logistic regression  Cox regression |
| Zhang | China (219 prefecture cities) | January 24-Febr. 29 | New daily cases | Temperature, relative humidity, wind  Air Quality index | Province, country-level daily new confirmed cases | Linear regression |
| Zhu L | China (120 cities) | January 23 – Febr. 29 | New daily cases | PM_2.5_, PM_10_, SO_2_, CO, NO_2_, O_3_ | Temperature, relative humidity, air pressure, wind speed | GAM – Gaussian link |
| Zhu Y | South America (regions of Ecuador, Brazil, Peru and Chile) | February 23 – May 6 | New daily cases  Daily incubative cases Reproductive number | Temperature, relative humidity, wind speed, visibility, absolute humidity |  | Linear regression |

**Table S3.- List of studies included in the qualitative synthesis.**

**Meteorological variables only**

Adenkule IA, Tella SA, Oyesiku KO, Oseni IO. Spatio-temporal analysis of meteorological factors in abating the spread of COVID-19 in Africa. *Heliyon.* 2020; 6(8):e04749. doi: <10.1016/j.heliyon.2020.e04749>.

Ahmadi M, Sharifi A, Dorosti S, Ghoushchi SJ, Ghanbari N. Investigation of effective climatoloty parameters on COVID-19 outbreak in Iran. *Sci Total Environ.* 2020; 729:138705. doi: <10.1016/j.scitotenv.2020.138705>.

Behnood A, Golafshani EM, Hosseini SM. Determinants of the infection rate of the COVID-19 in the U.S. using ANFIS and virus optimization algorithm (VOA). *Chaos Solitons Fractals.* 2020; 139:110051. doi: <10.1016/j.chaos.2020.110051>.

Briz-Redón A, Serrano-Aroca A. A spatio-temporal analysis for exploring the effect of temperature on COVID-19 early evolution in Spain. *Sci Total Environ.*  2020; 728:138811. doi: <10.1016/j.scitotenv.2020.138811>.

Byass P. Eco-epidemiological assessment of the COVID-19 epidemic in China, January-February 2020. *Glob Health Action.* 2020; 13(1):1760490. doi: <10.1080/16549716.2020.1760490>.

Carleton T, Cornetet J, Huybers P, Meng KC, Proctor J. Global evidence for ultraviolet radiation decreasing COVID-19 growth rates. *Proc Natl Acad Sci USA.* 2021 (updated Jan 7, 2021); 118(1):e2012370118. doi: <10.1073/pnas.2012370118>.

Chien LC, Chen LW. Meteorological impacts on the incidence of COVID-19 in the U.S. *Stoch Environ Res Risk Assess.*  2020; 4:1-6. doi: <10.1007/s00477-020-01835-8>.

Demongeot J, Flet-Berliac Y, Seligmann H. Temperature decreases spread parameters of the new COVID-19 case dynamics. *Biology (Basel).* 2020; 9(5):94. doi: <10.3390/biology9050094>.

Falcão-Sobral MF, da Penha Sobral AIG, Marinho MLM, de Souza Melo A. Associaction between climate variables and global transmission of SARS-CoV-2. *Sci Total Environ.* 2020: 729:138997. doi: <10.1016/j.scitotenv.2020.138997>.

Fazzini M, Baresi C, Bisci C, Bna C, Cecili A, Giuliacci A, Illuminatii S, Pregliasco F, Miccadei E. Preliminary analysis of relationships between COVID-19 and climate, morphology and urbanization in the Lombardy Region (Northern Italy). *Int J Environ Res Public Health.* 2020; 17(19):6955. doi: <10.3390/ijerph17196955>.

Fu S, Wang B, Zhou J, Xu X, Liu J, Ma U, Li L, He X, Li S, Niu J, Luo B, Zhang K. Meteorological factors, governmental responses and COVID-19: Evidence from four European countries. *Environ Res.* 2021 (Epub 2020 Dec 9); 194:110596. doi: <10.1016/j.envres.2020.110596>.

Guasp M, Laredo C, Urra X. Higher solar irradiance is associated with a lower incidence of coronavirus disease 2019. *Clin Infect Dis.* 2020; 71(16):2269-2271. doi: <10.1093/cid/ciaa575>.

Guo C, Bo Y, Changqing L, LI HB, Zeng Y, Zhang Y, Hossain S, Chan JWM, Yeung DW, Kwok KO, Wong SYS, Lau AKH, Lao XQ. Meteorological factors and COVID-19 incidence in 190 countries: An observational study. *Sci Total Environ.* 2021 (Epub 2020 Nov 23); 757:143783. doi: <10.1016/j.scitotenv.2020.143783>.

Guo XJ, Zhang H, Zeng YP. Transmissibility of COVID-19 in 11 major cities in China and its association with temperature and humidity in Beijing, Shanghai, Guangzhou, and Chengdu. *Infect Dis Poverty.* 2020; 9(1):87. doi: <10.1186/s40249-020-00708-0>.

He Z, Chin Y, Yu S, Huang J, Zhang CJP, Zhu K, Azarakhsh N, Sheng J, He Y, Jayavanth P, Liu Q, Akinwunmi BO, Ming WK. The influence of average temperatue and relative humidity on new cases of COVID-19: Time-series analysis. *JMIR Public Health Surveill.* 2021 (Jan 25, 2021); 7(1):e20495. doi: <10.2196/20495>.

Iqbal MM, Abid I, Hussain S, Shahzad N, Waqas MS, Iqbal MJ. The effects of regional climatic condition on the spread of COVID-19 at global scale. *Sci Total Environ.* 2020; 739:140101. doi: <10.1016/j.scitotenv.2020.140101>.

Iqbal N, Fareed Z, Shahzad F, He X, Shahzad U, Lina M. The nexus between COVID-19, temperature and exchange rate in Wuhan city: New findings from partial and multiple wavelet coherence. *Sci Total Environ.* 2020; 729:138916. doi: <10.1016/j.scitotenv.2020.138916>.

Isaia G, Diémoz H, Maluta F, Fountoulakis I, Ceccon D, di Sarra A, Facta S, Fedele F, Lorenzetto G, Siani AM, Isaia G. Does solar ultraviolet radiation play a role in COVID-19 infection and deaths? An environmental ecological study in Italy. *Sci Total Environ.* 2021 (Epub 2020 Nov 20); 757:143757. doi: <10.1016/j.scitotenv.2020.143757>.

Islam ART, Hasanuzzamaan Md, Shammi M, Salam R, Bodrud-Doza Md, Rahman, MM, Mannan MA, Huq S. Are meterological factor enhacing COVID-19 transmission in Bangladesh? Novel findings from a compound Poisson generalized linear modeling approach. *Environ Sci Pollut Res Int.* 2021 (Epub 2020 Oct 28); 28(9):11245-11258. doi: <10.1007/s11356-020-11273-2>.

Islam N, Bukhari Q, Jameel Y, Shabnam S, Erzurumluoglu AM, Siddique MA, Massaro JM, D’Agostino RB. COVID-19 and climatic factors: A global analysis. *Environ Res.* 2021 (Epub 2020 Oct 28); 193:110355. doi: <10.1016/j.envres.2020.110355>.

Jüni P, Rothenbühler M, Bobos P, Thorpe KE, da Costa BR, Fisman DN, Slutsky AS, Gesink D. impact of climate and public health interventions on the COVID-19 pandemic: a prospective cohort study. *CMAJ.* 2020: 192(21):E566-E573. doi: <10.1503/cmaj.200920>.

Khan IM, Haque U, Zhang W, Zafar S, Wang Y, He J, Sun H, Lubinda J, Rahman MS. COVID-19 in China: Risk factors and R_0_ revisited. *Acta Trop.* 2021 (Epub 2020 Oct 22); 213:105731. doi: <10.1016/j.actatropica.2020.105731>.

Lhada N, Bhardwaj P, Charan J, Mitra P, Goyal JP, Sharma P, Singh K, Misra S. Association of environmental parameters with COVID-19 in Delhi, India. *Indian J Clin Biochem.* 2020; 35(4):1-5. doi: <10.1007/s12291-020-00921-6>.

Lin J, Huang W, Wen M, Li D, Ma S, Hua J, Hu H, Yin S, Qian Y, Chen P, Zhang Q, Yuan N, Sun S. Containing the spread of coronavirus disease 2019 (COVID-19): Meteorological factors and control strategies. *Sci Total Environ.* 2020; 744:140935. doi: <10.1016/j.scitotenv.2020.140935>.

Liu J, Zhou J, Yao J, Zhang X, Li L, Xiaocheng X, He W, Wang B, Fu S, Niu T, Yan J, Shi Y, Ren X, Niu J, Zhu W, Li S, Luo B, Zhang L. Impact of meterological factors on the COVID-19 transmission: A multi-ciy study in China. *Sci Total Environ.* 2020: 726:138513. doi: <10.1016/j.scitotenv.2020.138513>.

Malki Z, Atlam ES, Hassanien AE, Dagnew G, Elhosseini MA, Gad I. Association between weather data and COVID-19 pandemic predicting mortality rate: Machine learning approaches. *Chaos Solitons Fractals.* 2020; 138:110137. doi: <10.1016/j.chaos.2020.110137>.

Mandal CC, Panwar MS. Can the summer temperatures reduce COVID-19 cases? *Public Health.* 2020; 185:72-79. doi: <10.1016/j.puhe.2020.05.065>.

Medeiros- Figueiredo A, Daponte-Codina A, Moreira Marculino Figueiredo DC, Toledo Vianna RP, Costa de Lima K, Gil-Garceia E. Factors associated with the incidence and mortality from COVID-19 in the autonomous communities of Spain [in Spanish]. *Gac Sanit.* 2020; 30:S0213-9111(20)30124-2. doi: <10.1016/j.gaceta.2020.05.004>.

Meo SA, Abukhalaf AA, Alomar AA, Al-Beeshi IZ, Alhowikan A, Shafi KM, Meo AS, Usmani AM, Akram J. Climate and COVID-19 pandemic: Effect of heat and humidity on the incidence and mortality in world’s top ten hottest and top ten coldest countries. *Eur Rev Med Pharmacol Sci.* 2020; 24(15):8232-8238. doi: <10.26355/eurrev_202008_22513>.

Meraj G, Farooq M, Singh SK, Romshoo SA, Sudhanshu, Nathawat MS, Kanga S. Coronavirus pandemic versus temperature in the context of Indian subcontinent: a preliminary statisticsl analsys. *Environ Dev Sustain.* 2020; 1-11. doi: <10.1007/s10668-020-00854-3>.

Meyer A, Sadler R, Faverjon C, Cameron AR, Bannister-Tyrrell M. Evidence that higher temperature are associated with a marginally lower incidence of COVID-19 cases. *Front Public Health.* 2020: 8:367. doi: <10.3389/fpubh.2020.00367>.

Muñoz-Cacho, Hernández JL, López-Hoyos M, Martínez-Taboada VM. Can climatic factors explain the differences in COVID-19 incidence and severity across the Spanish regions?: An ecological study. *Public Health.* 2020; 19(1):106. doi: <10.1186/s12940-020-00660-4>.

Notari A. Temperature dependence of COVID-19 transmission. *Sci Total Environ.* 2021 (Epub 2020 Dec 13); 763:144390. doi: <10.1016/j.scitotenv.2020.144390>.

Ozygit A. Understanding COVID-19 transmission: The effect of temperature and health behavior on transmission rates. *Infect Dis Health.* 2020; 25(4):233-238. doi: <10.1016/j.idh.2020.07.001>.

Paez A, López FA, Menezes T, Cavalcanti R, Pitta MGDR. A spatio-temporal analysis of the environmental correlates of COVID-19 incidence in Spain. *Geogr Anal.* 2020; 8:10.1111/gean.12241. doi: <10.1111/gean.12241>.

Pan J, Yao Y, Liu Z, Meng X, Ji JS, Qiu Y, Wang W, Zhang L, Wang W, Kan H. Warmer weather unlikely to reduce the COVID-19 transmission: An ecological study in 202 locations in 8 countries. *Sci Total Environ.* 2021 (Epub 2020 Sep 9); 753:142272. doi: <10.1016/j.scitotenv.2020.142272>.

Pequeno P, Mendel B, Rosa C, Bosholn M, Souza JL, Baccaro F, Barbosa R, Magnusson W. Air transportation, population density and temperature predict the spread of COVID-19 in Brazil. *PeerJ* 2020: 8:e9322. doi: <10.7717/peerj.9322>.

Pirouz B, Shaffiee Haghshenas S, Pirouz B, Shaffiee Haghshenas S, Piro P. Development of an assessment method for investigatint the impact of climate and urban parameters in confirmed cases of COVID-19: A new challenge in sustainable development. *Int J Environ Res Public Health.* 2020; 17(8):2801. doi: <10.3390/ijerph17082801>.

Poirier C, Luoo W, Majumder MS, Liu D, Mandl KD, Mooring TA, Santillana M. The role of environmental factors on transmission rates of the COVID-19 outbreak: an initial assessment in two spatial scales. *Sci Rep.* 2020; 10(1):17002. doi: <10.1038/s41598-020-74089-7>.

Pramanik M, Udmale P, Bisht P, Chowdhury K, Szabo S, Pal I. Climatic factors influence the spread of COVID-19 in Russia. *Int J Environ Health Res.* 2020; 16:1-15. doi: <10.1080/09603123.2020.1793921>.

Prata DN, Rodrigues W, Bermejo PH. Temperature significantly changes COVID-19 transmission in (sub)tropical cities of Brazil. *Sci Total Environ.* 2020; 729:138862. doi: <10.1016/j.scitotenv.2020.138862>.

Qi H, Xiao S, Shi R, Ward MP, Chen Y, Tu W, Su Q, Wang W, Wang X, Zhang Z. COVID-19 transmission in Mainland China is associated with temperature and humidity: A time-series analysis. *Sci Total Environ.* 2020; 728:138778. doi: <10.1016/j.scitotenv.2020.138778>. .

Rashed EA, Kodera S, Gomez-Tames J, Hirata A. Influence of absolute humidity, temperature and population density on COVID-19 spread and decay durations: Multi-prefecture study in Japan. *Int J Environ Res Public Health.* 2020; 17(15):5354. doi: <10.3390/ijerph17155354>.

Rehman Y, Rehman N. Association of climatic factors with COVID-19 in Pakistan. *AIMS Public Health.* 2020; 7(4):854-868. doi: <10.3934/publichealth.2020066>.

Rubin D, Huang J, Fisher BT, Gasparrini A, Tam V, Song L, Wang X, Kaufman J, Fitzpatrick K, Jain A, Griffis H, Cramer K, Morris J, Tasian G. Association of social distancing, population density, and temperature with the instantaneous reproduction number of SARS-CoV-2 in counties across the United States. *JAMA Netw Open.* 2020; 3(7):e2016099. doi: <10.1001/jamanetworkopen.2020.16099>.

Runkle JD, Sugg MM, Leeper RD, Rao Y, Matthews JL, Rennie JJ. Short-term effects of specific humidity and temperature on COVID-19 morbidity in select US cities. *Sci Total Environ.* 2020; 740:140093. doi: <10.1016/j.scitotenv.2020.140093>.

Sajadi MM, Habibzadeh P, Vintzileos A, Shokouhi S, Miralles-Wilhelm F, Amoroso A. Temperature, humidity, and latitude analysis to estimate potential spread and seasonality of Coronavirus Disease 2019 (COVID-19). *JAMA Netw Open.* 2020; 3(6):e2011834. doi: <10.1001/jamanetworkopen.2020.11834>.

Sánchez-Lorenzo A, Vaquero-Martínez J, Calbó J, Wild M, Santurtún A, López-Bustins JA, Vaquero JM, Folini D, Antón M. Dis anomalous atmospheric circulation favor the spread of COVID-19 in Europe? *Environ Res.* 2021 (Epub 2020 Dec 17); 194:110626. doi: <10.1016/j.envres.2020.110626>.

Sarkodie SA, Owusu PA. Impact of meteorological factors on COVID-19 pandemic: Evidence from top 20 countries with confirmed cases. *Environ Res.* 2020; 191:110101. doi: <10.1016/j.envres.2020.110101>.

Sehra ST, Salciccioli JD, Wiebe DJ, Fundin S, Baker JF. Maximum daily temperature, precipitation, ultraviolet light, and rates of transmission of severe acute respiratory syndrome Coronavirus 2 in the United States. *Clin Infect Dis.* 2020; 71(9):2482-2487. doi: <10.1093/cid/ciaa681>.

Shahzad F, Shahzad U, Fareed Z, Iqbal N, Hashmi SH, Ahmad F. Asymmetric nexus between temperature and COVID-19 in the top ten affected provinces of China: A current application of quantile-on-quantile approach. *Sci Total Environ.* 2020; 736:139115. doi: <10.1016/j.scitotenv.2020.139115>.

Shao W, Xie J, Zhu Y. Mediation by human mobility of the association between temperature and COVID-10 transmission rate. *Environ Res.* 2021 (Epub 2020 Dec 16); 194:110608. doi: <10.1016/j.envres.2020.110608>.

Shi P, Dong Y, Yan H, Zhao C, Li X, Liu W, He M, Tang S, Xi S. Impact of temperature on the dynamics of the COVID-19 outbreak in China. *Sci Total Environ.* 2020; 728:138890. doi: <10.1016/j.scitotenv.2020.138890>.

Tagaki H, Kuno T, Yokoyama Y, Ueyama H, Matsushiro T, Hari Y, Ando T a. The higher temperature and ultraviolet, the lower COVID-19 prevalence-meta-regression of data from large US cities. *Am J Infect Control.* 2020; 48(10):1281-1285. doi: <10.1016/j.ajic.2020.06.181>.

Tagaki H, Kuno T, Yokoyama Y, Ueyama H, Matsushiro T, Hari Y, Ando T b. Higher temperature, pressure, and ultraviolet are associated with less COVID-19 prevalence: Meta-regression of Japanese prefectural data. *Asia Pac J Public Health.* 2020; 32(8):520-522. doi: <10.1177/1010539520947875>.

To T, Zhang K, Maguire B, Terebessy E, Fong I, Parikh S, Zhu J. Correlation of ambient temperature and COVID-19 incidence in Canada. *Sci Total Environ.* 2012 (Epub 2020 Aug 4); 759:141484. doi: <10.1016/j.scitotenv.2020.141484>.

Tobías A, Molina T. Is temperature reducing the transmission of COVID-19? *Environ Res.* 2020; 186:109553. doi: <10.1016/j.envres.2020.109553>.

Tzampoglou P, Loukidis D. Investigation of the importance of climatic factors in COVID-19 worldwide intensity. *Int J Environ Res Public Health.* 2020; 17(21):7730. doi: <10.3390/ijerph17217730>.

Ujiie M, Tsuzuki S, Ohmagari N. Effect of temperature on the infectivity of COVID-19. *Int J Infect Dis.* 2020; 95:301-303. doi: <10.1016/j.ijid.2020.04.068>.

Wang Q, Zhao Y, Zhang Y, Qiu J, Li J, Yan N, Li N, Zhang J, Tian D, Sha X, Jing J, Yang C, Wang K, Xu R, Zhang Y, Yang H, Zhao S, Zhao Y. Could the ambient higher temperature decrease the transmissibility of COVID-19 in China? *Environ Res.* 2021 (Epub 2020 Dec 3); 193:110576. doi: <10.1016/j.envres.2020.110576>.

Ward MP, Xiao S, Zhang Z a. The role of climate during the COVID-19 epidemic in New South Wales, Australia. *Transbound Emerg Dis.* 2020; 67(6):2313-2317. doi: <10.1111/tbed.13631>.

Ward MP, Xiao S, Zhang Z b. Humidity is a consistent climatic factor contributing to SARS-CoV-2 transmission. *Transbound Emerg Dis.* 2020; 67(6):3069-3074. doi: <10.1111/tbed.13766>.

Wu Y, Jing W, Liu J, Ma Q, Yuan J, Wang Y, Du M, Liu M. Effects of temperature and humidity on the daily new cases and new deaths of COVID-19 in 166 countries. *Sci Total Environ.* 2020; 729:139051. doi: <10.1016/j.scitotenv.2020.139051>.

Xie J, Zhu Y. Association between ambient temperature and COVID-19 infection in 122 cities from China. *Sci Total Environ.* 2020; 724:138201. doi: <10.1016/j.scitotenv.2020.138201>.

Yao Y, Pan J, Liu Z, Meng X, Wang W, Kan H, Wang W a. No association of COVID-19 transmission with temperature or UV radiation in Chinese cities. *Eur Respir J.* 2020; 55(5):2000517. doi: <10.1183/13993003.00517-2020>.

Zhang Z, Xue T, Jin X. Effects of meteorological conditions and air pollution on COVID-19 transmission: Evidence from 219 Chinese cities. *Sci Total Environ.* 2020; 741:140244. doi: <10.1016/j.scitotenv.2020.140244>.

Zhu L, Liu X, Huang H, Avellán-Llaguno RD, Llaguno-Lazo M, Gaggero A, Soto-Rifo R, Patiñooo L, Valencia-Avellán M, Diringer B, Huang Q, Zhu YG. Meteorological impact on the COVID-19 pandemic: A study across eight severely affected regions in South America. *Sci Total Environ.* 2020; 744:140881. doi: <10.1016/j.scitotenv.2020.140881>.

**Air pollutants only**

Aabed K, Lashin MMA. An analytical study of the factors that influence COVID-19 spread*. Saudi J Biol Sci.* 2021 (Epub 2020 Nov 26); 28(2):1177-1195. doi: <10.1016/j.sjbs.2020.11.067>.

Chakrabarty RK, Beeler P, Liu P, Gooswami S, Harvey RD, Pervez S, van Donkelaar A, Martin RV. Ambient PM_2.5_ exposure and rapid spread of COVID-19 in the United States. *Sci Total Environ.* 2021 (Epub 2020 Nov 9); 760:143391. doi: <10.1016/j.scitotenv.2020.143391>.

Fattorini D, Regoli F. Role of the chronic air pollution levels in the COVID-19 oubreak risk in Italy. *Environ Pollut.* 2020; 264:114732. doi: <10.1016/j.envpol.2020.114732>.

Fiasca F, Minelli M, Maio D, Minelli M, Vergallo I, Necozione S, Mattei A. Associations between COVID-19 incidence rates and the exposure to PM2.5 and NO2: A nationwide observational study in Italy. *Int J Environ Res Public Health.* 2020; 17(24):9318. doi: <10.3390/ijerph17249318>.

Filippini T, Rothman KJ, Goffi A, Ferrari F, Maffeis G, Orsini N, Vinceti M. Satellite-detected trophospheric nitrogen dioxide and spreadd of SARS-CoV-2 infection in Northern Italy. *Sci Total Environ.* 2020, 739:140278. doi:  <10.1016/j.scitotenv.2020.140278>.

Gupta A, Bherwani H, Gautam S, Anjum S, Musugu K, Kumar N, Anshul A, Kumar R. Air pollution aggravating COVID-19 lethality? Exploration in Asian cities using statistical models. *Environ Dev Sustain*. 2020; 15:1-10. doi: <10.1007/s10668-020-00878-9>.

Hoang T, Nguyen TQ, Tran TTA b. Short-term exposure to ambient air pollution in association with COVID-10 of two clusters in South Korea. *Trop Med Int Health*. 2021 (Epub 2021 Jan 17); 36(4):478-491. doi: <10.1111/tmi.13538>.

Hutter HP, Poteser M, Moshammer H, Lemmerer K, Mayer M, Weitensfelder L, Wallner P, Kundi M. Air pollution is associated with COVID-19 incidence and mortality in Vienna, Austria. *Int J Environ Res Public Health.* 2020; 17(24):9275. doi: <10.3390/ijerph17249275>.

Liang D, Shi L, Zhao J, Liu P, Sarnat JA, Gao S, Schwartz J, Liu Y, Ebelt ST, Scovronick N, Chang HH. Urban air pollution may enhace COVID-19 case-fatality and mortality rates in the United States. *Innovation (NY).* 2020; 1(3):100047. doi: <10.1016/j.xinn.2020.100047>.

López-Feldman A, Heres D, Márquez-Padilla F. Air pollution exposure and COVID-19: A look at mortality in Mexico City using individual-level data. *Sci Total Environ.* 2021 (Epub 2020 Nov 26); 756:143929. doi: <10.1016/j.scitotenv.2020.143929>.

Pozzer A, Dominici F, Haines A, Witt C, Münzel T, Lelieveld J. Regional and global contributions of air pollution to risk of death from COVID-19. *Cardiovasc Res.* 2020; 116(14):2247-2253. doi: <10.1093/cvr/cvaa288>.

Wang Y, Di Q. Modifiable areal unit problem and environmental factors of COVID-19 outbreak. *Sci Total Environ.* 2020; 740:139984. doi: <10.1016/j.scitotenv.2020.139984>.

Yao Y, Pan J, Liu Z, Meng X, Wang W, Kan H, Wang W b. Ambient nitrogen dioxide pollution and spreadbility of COVID-19 in Chinese cities. *Ecotoxicol Environ Saf.* 2021 (Epub 2020 Sep 30); 208:111421. doi: <10.1016/j.ecoenv.2020.111421>.

Zhu Y, Xie J, Huang F, Cao L. Association between short-term exposure to air pollution and COVID-19 infection: Evidence from China. *Sci Total Environ.* 2020; 727:138704. doi: <10.1016/j.scitotenv.2020.138704>.

**Socioeconomic variables only**

Azar KMJ, Shen Z, Romanelli RJ, Lockhart SH, Smits K, Robinson S, Brown S, Pressman AR. Disparities in outcomes among COVID-19 patients in a large health care system in California. *Health Aff (Millwood)* 2020; 39(7):1253-1262. doi: <10.1377/hlthaff.2020.00598>.

Chakraborty J. Convergence of COVID-19 and chronic air pollution risks: Racial/ethnic and socioeconomic inequities in the U.S. *Environ Res.* 2021 (Epub 2020 Dec 10); 193:110586. doi: <10.1016/j.envres.2020.110586>.

Chaudhry R, Dranitsaris G, Mubashir T, Bartoszko J, Riazi S. A country level analysis measuring the impact of government actions, country preparedness and socioeconomic factors on COVID-19 mortality and related health outcomes. *EClinicalMedicine.* 2020; 25:100464. doi: <10.1016/j.eclinm.2020.100464>.

Das A, Ghosh S, Das K, Basu T, Das M, Dutta I a. Modeling the effect of area deprivation on COVID-19 incidences: a study of Chennai megacity, India. *Public Health.* 2020; 185:266-269. doi: <10.1016/j.puhe.2020.06.011>.

Das A, Ghosh S, Das L, Basu T, Dutta I, Das M b. Living environment matters: Unravelling the spatial clustering of COVID-19 hotspots in Kolkata megacity, India. *Sustain Cities Soc.* 2021 (Epub 2020 Oct 31): 65:102577. doi: <10.1016/j.scs.2020.102577>.

DiMaggio C, Klein M, Berry C, Frangos S. Black/African American Communities are at highest risk of COVID-19: spatial modeling of New York City ZIP Code-level testing results. *Ann Epidemiol.* 2020; 51:7-13. doi: <10.1016/j.annepidem.2020.08.012>.

Drefahl S, Wallace M, Mussino E, Aradhya S, Kolk M, Brandén M, Malmberg B, Andersson G. A population-based cohort study of socio-demographic risk factors for COVID-19 deaths in Sweden. *Nat Commun.* 2020; 11(1):5097. doi: <10.1038/s41467-020-18926-3>.

Kaiser JC, Stathopoulos GT. Socioeconomic correlates of SARS-CoV-2 and influenza H1N1 outbreaks. *Eur Respir J.* 2020; 56(3):2001400. doi: <10.1183/13993003.01400-2020>.

Lamb MR, Kandula S, Shaman J. Differential COVID-19 case positivity in New York City neighborhoods: Socioeconomic factors and mobility. *Influenza Other Respir Viruses.* 2021 (Epub 2020 Oct 14); 15(2):209-217. doi: <10.1111/irv.12816>.

Lau H, Khosrawipour V, Kocbach P, Mikolajczyk A, Ichii H, Zacharski M, Bania J, Khosrawipour T. The association between international and domestic air traffic and the coronavirus (COVID-19) outbreak. *J Microbiol Immunol Infect.* 2020; 53(3):467-472. doi: <10.1016/j.jmii.2020.03.026>.

Li X, Zhou L, Jia T, Peng R, Fu X, Zou Y. Associating COVID-19 severity with urban factors: A case study of Wuhan. *Int J Environ Res Public Health.* 2020; 17(18):6712. doi: <10.3390/ijerph17186712>.

Madhav KC, Oral E, Straif-Bourgeois S, Rung AL, Peters ES. The effect of area deprivation on COVID-19 risk in Lousiana. *PLoS One.* 2020; 15(12):e0243028. doi: <10.1371/journal.pone.0243028>.

Marciel de Souza W, Fletcher Buss L, da Silva Candido D, Carrera JP, Li S, Zarebski AE, Moraes Pereira Rh, Prete CA, de Souza-Santos AA, Parag KV, Belotti MCTDD, Vincenti-González MF, Messina J, da Silva Sales FC, Dos Santos Andrade P, Heloiz Nascimento V, Ghilardi F, Abade L, Gutiérrez B, Kraemer MUG, Braga CKV, Santana Aguiar R, Alexander N, Mayaud p, Brady OJ, Marcilio I, Gouveia N, Li G, Tami A, Barbosa de Oliveira S, Gomes Porto VB, Ganem F, Ferreira de Almeida WA, Sutile Tardetti Fantinato FF, Marques Macário E, Kleber de Oliveiira W, Nogueiira ML, Pybus OG, Wu CH, Croda J, Sabino EC, Rodrigues Faria N. Epidemiological and clinical characteristics of the COVID-10 epidemic in Brazil. *Nat Hum Behav.* 2020; 4(8):856-865. doi: <10.1038/s41562-020-0928-4>.

Plümper T, Neumayer E. The pandemic predominantly hits poor neighboourhoods? SARS-CoV-2 infections and COVID-19 fatalities in German districts. *Eur J Public Health.* 2020; 30(6):1176-1180. doi: <10.1093/eurpub/ckaa168>.

Price-Haywood EG, Burton H, Fort D, Seoane L. Hospitalization and mortality among Black patients and White patients with COVID-19. *N Engl J Med.* 2020; 382(26):2534-2543. doi: <10.1056/NEJMsa2011686>.

Rafael RMR, Neto M, Depret DG, Gil AC, Fonseca MHS, Souza-Santos R. Effect of income on the cumulative incidence of COVID-19: an ecological study. *Rev Lat Am Enfermagem.* 2020; 28:e3344. doi: <10.1590/1518-8345.4475.3344>.

Richmond Hl, Tome J, Rochani H, Fung CH, Shah GH, Schwind JS. The use of penalized regression analysis to identify county-level demographic and socioeconomic variables predictive of increased COVID-19 cumulative case rates in the state of Georgia. *Int J Environ Res Public Health.* 2020; 17(21):8036. doi: <10.3390/ijerph17218036>.

Rozenfeld Y, Beam J, Maier H, Haggerson W, Boudreau K, Carlson j, Medows R. A model of disparities: risk factors associated with COVID-19 infection. *Int J Equity Health.* 2020; 19(1):126. doi: <10.1186/s12939-020-01242-z>.

Sannigrahi S, Pilla F, Basu B, Basu AS, Molter A. Examining the association between socio-demographic composition and COVID-29 fatalities in the European region using spatial regression approach. *Sustain Cities Soc.* 2020; 62:102418. doi: <10.1016/j.scs.2020.102418>.

Scarpone C, Brinkmann ST, Große T, Sonnenwald D, Fuchs M, Byron Walker B. A multimethod approach for county-scale geospatial analysis of emerging infectious diseases: a cross-sectional case study of COVID-19 incidence in Germany. *Int J Health Geogr.* 2020; 19(1):32. doi: <10.1186/s12942-020-00225-1>.

You H, Wu X, Guo X. Distribution of COVID-19 morbidity rate in association with social and economic factors in Wuhan, China: Implications for urban development. *Int J Environ Res Public Health.* 2020; 17(10):3417. doi: <10.3390/ijerph17103417>.

Zakery R, Bendayan R, Ashworth M, Bean DM, Dodhia H, Durbaba S, O’Gallagher K, Palmmer C, Curcin V, Aitken E, Bernal W, Barker RD, Norton S, Gulliford m, Teo JTH, Galloway J, Dobson RJB, Shah AM. A case-control and cohort study to determine the relationship between ethnic background and severe COVID-19. *EClinicalMedicine.* 2020; 28:100574. doi: <10.1016/j.eclinm.2020.100574>.

**Meteorological variables and air pollutants**

Adhikari A, Yin J. Short-term effects of ambient ozone, PM_2.5_, and meteorological factors on COVID-19 confirmed cases and deaths in Queens, New York. *Int J Environ Res Public Health.* 2020; 17(11):4047. doi: <10.3390/ijerph17114047>.

Azuma K, Kagi N, Kim H, Hayashi M. Impact of climate and ambient air pollution on the epidemic growth during COVID-19 outbreak in Japan. *Environ Res.* 2020; 190:110042. doi: <10.1016/j.envres.2020.110042>.

Coccia M b. How do low wind speeds and high levels of air pollution support the spread of COVID-19? *Atmos Pollut Res.* 2021 (Epub 2020 Oct 7); 12(1):437-445. doi: <10.1016/j.apr.2020.10.002>.

Dogan B, Jebli MB, Shahzad K, Farooq TH, Shahzad U. Investigating the effects of meteorological parameters on COVID-19: Case study of New Jersey, United States. *Environ Res.* 2020; 191:110148. doi: <10.1016/j.envres.2020.110148>.

Hoang T, Tran TT a. Ambient air pollution, meterology, and COVID-19 infection in Korea. *J Med Virol.* 2021 (Epub 2020 Jul 27); 93(2):878-885. doi: <10.1002/jmv.26325>.

Jiang Y, Wu XJ, Guan YJ. Effect of ambient air pollutants and meteorological variables on COVID-19 incidence. *Infect Control Hosp Epidemiol.* 2020; 41(9):1011-1015. doi: <10.1017/ice.2020.222>.

Li H, Xu XL, Dai DW, Huang ZY, Ma Z, Guan YJ. Air pollution and temperature are associated with increased COVID-19 incidence: A time series study. *Int J Infect Dis.* 2020; 97:278-282. doi: <10.1016/j.ijid.2020.05.076>.

Ma Y, Zhao Y, Liu J, He X, Wang B, Fu S, Yan J, Niu J, Zhou J, Luo B. Effects of temperature variation and humididty on the death of COVID-19 in Wuhan, China. *Sci Total Environ.* 2020; 724:138226. doi: <10.1016/j.scitotenv.2020.138226>.

Martorell-Marugán J, Villatoro-García JA, García-Moreno A, López-Domínguez R, Requena F, Merelo JJ, Lacasaña M, Luna JD, Díaz-Mochón JJ, Lorente JA, Carmona-Saéz P. DatAC: A visual analytics platform to explore climate and air quality indicators associated with the COVID-19 pandemic in Spain. *Sci Total Environ.* 2021 (Epub 2020 Aug 4); 750:141424. doi: <10.1016/j.scitotenv.2020.141424>.

Shahzad K, Shahzad U, Shahzad F, Fareed Z. Effects of climatological parameters on the outbreak spread of COVID-19 in highly affected regions of Spain. *Environ Sci Pollut Res Int.* 2020; 27(31):39657-39666. doi: <10.1007/s11356-020-10551-3>.

Stieb DM, Evans GJ, To TM, Brook JR, Burnett RT. An ecological analysis of long-term exposure to PM_2.5_ and incidence of COVID-19 in Canadian health regions. *Environ Res.* 2020; 191:110052. doi: <10.1016/j.envres.2020.110052>.

**Meteorological and socioeconomic variables**

Jamshidi S, Baniasad M, Niyogi D. Global to USA county scale analysis of weather, urban density, mobility, homestay, and mask use on COVID-19. *Int J Environ Res Public Health.* 2020; 17(21)7847. doi: <10.3390/ijerph17217847>.

Kodera S, Rashed EA, Hirata A. Correlation between COVID-19 morbidity and mortality rates in Japan and local population density, temperature, and absolute humidity. *Int J Environ Res Public Health.* 2020; 17(15):5477. doi: <10.3390/ijerph17155477>.

Kubota Y, Shiono T, Kusumoto B, Fujinuma J. Multiple drivers of the COVID-19 spread: The roles of climate, international mobility, and region-specific conditions. *PLoS One.* 2020; 15(9):e0239385. doi: <10.1371/journal.pone.0239385>.

Li AY, Hannah TC, Durbin JR, Dreher N, McAuley FM, Fares Marayati N, Spiera Z, Ali M, Gometz A, Kostman JR, Choudhri TF. Multivariate analysis of black race and environmental temperature on COVID-19 in the US. *Am J Med Sci.* 2020; 360(4):348-356. doi: <10.1016/j.amjms.2020.06.015>.

Luo Y, Yan j, McClure S. Distribution of the environmental and socioeconomic risk factors on COVID-19 death rate across continental USA: a spatial nonlinear analysis. *Environ Sci Pollut Res Int.* 2021 (Epub 2020 Oct 1); 28(6).6587-6599. doi: <10.1007/s11356-020-10962-2>.

Su M, Peng S, Chen L, Wang B, Wang Y, Fan X, Dong Z. A warm summer is unlikely to stop transmission of COVID-19 naturally. *Geohealth.* 2020; 4(12):e2020GH000292. doi: <10.1029/2020GH000292>.

Sun Y, Hu X, Xie J. Spatial inequalities of COVID-10 mortality rate in relation to socioeconomic and environmental factors across England. *Sci Total Environ.* 2021 (Epub 2020 Nov 13); 758:143595. doi: <10.1016/j.scitotenv.2020.143595>.

Xie Z, Qin Y, Li Y, Shen W, Zheng Z, Liu S. Spatial and temporal differentiation of COVID-10 epidemic spread in mainland China and its influencing factors. *Sci Total Environ.* 2020; 744:140929. doi: <10.1016/j.scitotenv.2020.140929>.

Xu H, Yan C, Fu Q, Xiao K, Yu Y, Han D, Wang W, Cheng J. Possible environmental effects on the spread of COVID-19 in China. *Sci Total Environ.* 2020; 731:139211. doi: <10.1016/j.scitotenv.2020.139211>.

**Air pollutants and socioeconomic variables**

Chadeau-Hyam M, Bodinier B, Elliott J, Whitaker MD, Tzoulaki I, Vermeulen R, Kelly-Irving M, Delpierre C, Elliott P. Risk factors for positive and negative COVID-19 tests: a cautious and in-depth analysis of UK biobank data. *Int J Epidemiol.* 2020: 49(5):1454-1467. doi: <10.1093/ije/dyaa134>.

Han Y, Yang L, Jia K, Li J, Feng S, Chen W, Zhao W, Pereira P. Spatial distribution characteristics of the COVID-19 pandemic in Beijing and its relationship with environmental factors. *Sci Total Environ.* 2021 (Epub 2020 Dec 10); 761:144257. doi: <10.1016/j.scitotenv.2020.144257>.

Rodríguez-Villamizar L, Belalcázar-Ceróon LC, Fernández-Niño JA, Marín-Pineda DM, Rojas-Sánchez O, Acuña-Merchán LA, Ramírez-García N, Mangones-Matos SC, Vargas-González JN, Herrera-Torres J, Agudelo-Castañeda DM, Piñeros-Jiménez JG, Rojas-Roa NY, Herrera-Galindo M. Air pollution, sociodemographic and health conditions effects on COVID-19 mortality in Colombia: An ecological study. *Sci Total Environ.* 2021 (Epub 2020 Nov 26); 756:144020. doi: <10.1016/j.scitotenv.2020.144020>.

Saez M, Tobias A, Barceló MA. Effects of long-term exposure to air pollutants on the spatial spread of COVID-19 in Catalonia, Spain. *Environ Res.* 2020; 191:110177. doi: <10.1016/j.envres.2020.110177>.

**Meteorological variables, air pollutants and socioeconomic variables**

Coccia M a. Factors determining the diffusion of COVID-19 and suggested strategy to prevent future accelerated viral infectivity similar to COVID-19. *Sci Total Environ.* 2020; 729:138474. doi: <10.1016/j.scitotenv.2020.138474>.

Coker ES, Cavalli L, Fabrizi E, Guastella G, Lippo E, Parisi ML, Pontarollo N, Rizzati M, Varacca A, Vergalli S. The effects of air pollution on COVID-19 related mortality in Northern Italy. *Environ Resour Econ (Dordr).* 2020; 1-24. doi: <10.1007/s10640-020-00486-1>.

Perone G. The determinants of COVID-19 case fatality rate (CFR) in the Italian regions and provinces: An analysis of environmental, demographic, and health care factors. *Sci Total Environ.* 2021 (Epub 2020 Sep 24); 755(Pt 1):142523. doi: <10.1016/j.scitotenv.2020.142523>.

Rahman N, Islam M, Shimanto MH, Ferdous J, Shanto Rahman AAN, Sagor PS, Chowdhury T. A global analysis on the effect of temperature, socio-economic and environmental factors on the spread and mortality rate of the COVID-19 pandemic. *Environ Dev Sustain.* 2020; 1-15. doi: <10.1007/s10668-020-01028-x>.

Wu X, Nethery RC, Sabath MB, Braun D, Dominici F. Air pollution and COVID-19 mortality in the United States: Strengths and limitations of an ecological regression analysis. *Sci Adv.* 2020; 6(45):eabd4049. doi: <10.1126/sciadv.abd4049>.

**Figure S1.- Number of studies by type of explanatory variable analyzed.**


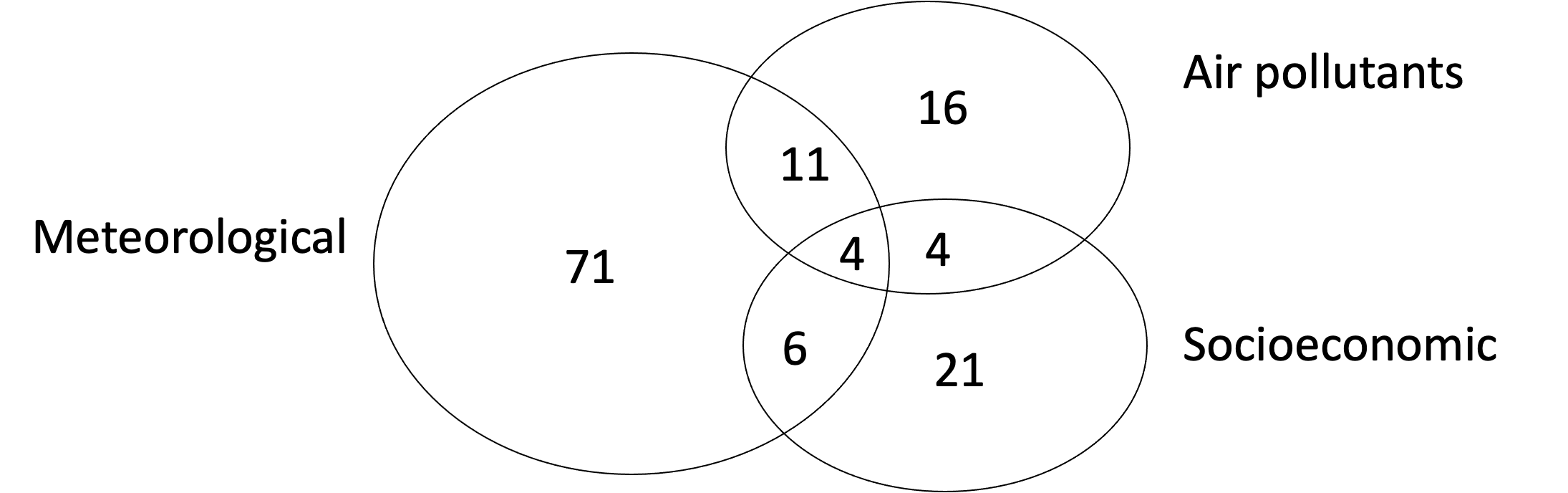

Supplement: Supplementary file 1 — Additional file 1: Table S1. List of studies excluded. Table S2. Studies included in the qualitative synthesis Table S3. List of studies included in the qualitative synthesis. Figure S1. Number of studies by type of explanatory variable analyzed [file 12302_2021_550_MOESM1_ESM.docx]
